# Mitogenomic Characterization and Phylogenetic Expansion of Tribe Coccinellini (Coleoptera: Coccinellidae)

**DOI:** 10.1002/ece3.73241

**Published:** 2026-03-12

**Authors:** Xin‐Yi Li, Zafar Iqbal, Fang Qi, Lin Xiaoling, Xing‐Min Wang, Rui‐E Nie

**Affiliations:** ^1^ The Anhui Provincial Key Laboratory of Biodiversity Conservation and Ecological Security in the Yangtze River Basin, College of Life Sciences Anhui Normal University Wuhu Anhui China; ^2^ Key Laboratory of Bio–Pesticide Innovation and Application, Engineering Technology Research Center of Agricultural Pest Biocontrol, Guangdong Province, Department of Entomology South China Agricultural University Guangzhou China

**Keywords:** Coccinellinae, Coccinellini, mitochondrial genome, phylogeny

## Abstract

Coccinellidae (Coleoptera) is a large and ecologically important beetle family, widely recognized for its members' role as natural enemies of agricultural pests. Within this family, the tribe Coccinellini is of particular significance because it includes many dominant predatory species used in biological control programs and exhibits notable ecological and trophic diversity. Despite their ecological and taxonomic importance, evolutionary relationships within Coccinellini remain unresolved because previous studies relied on limited molecular markers or morphology. In this study, we report nine complete mitochondrial genomes of Coccinellini species, estimated divergence time to place to their evolutionary diversification and present a comprehensive analysis of the features of these newly sequenced mitochondrial genomes. The newly sequenced mitogenomes contain the typical 37 genes (13 PCGs, two rRNAs, and 22 tRNAs) and a non‐coding control region, and are arranged in the same order as that of the putative ancestor of beetles. The average A + T content of all nine mitogenomes is 78.2%. The calculated values of relative synonymous codon usage (RSCU) determine codons UUA (L), UCU (S2), and CCU (P) have the highest frequency in all nine mitogenomes. Furthermore, combining 58 available mitogenomes, we reconstructed the tribe's phylogeny and resolved the taxonomic status of the studied species. Our phylogenetic analyses confirm the taxonomic placement of all the newly sequenced species within Coccinellini and support the monophyly of Coccinellini (Bootstrap support ≥ 70%, posterior probability ≥ 0.97). The tribe Coccinellini was resolved into four major clades with the following relationship: (Clade D, (Clade C, (Clade B, Clade A))). The mycophagous genera *Halyzia*, *Psyllobora*, *Illeis*, and *Vibidia* were consistently grouped within Coccinellini. These findings refine current understanding of evolutionary relationships within Coccinellini and provide a useful mitogenomic reference for future systematic related research in Coccinellidae.

## Graphical abstract

This study presents the complete mitochondrial genomes of nine ladybird species from the tribe Coccinellini and compares them with 58 previously published mitogenomes. Phylogenetic analyses confirm the taxonomic placement of these species within Coccinellini. The results support that the tribe Coccinellini is monophyletic with four main clades. Consistent grouping of several mycophagous genera and the discovery of novel relationships highlight both the complexity and the need for taxonomic revision within the group. The new mitochondrial data generated in this study will aid in species identification and more profound understanding of evolutionary relationships within the Coccinellidae.

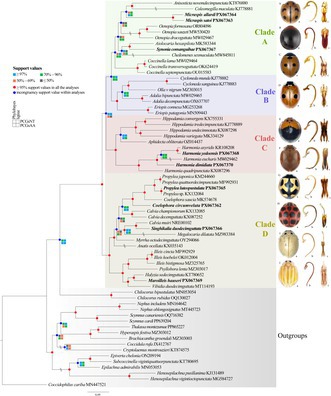

## 1 Introduction

Ladybird beetles are predatory on a wide range of insect pest species and are used as biological control agents (Hodek and Honěk 1996). There are approximately 6900 species of ladybird beetles (Ślipiński 2007; Vandenberg 2002), which belong to the superfamily Coccinelloidea (Insecta: Coleoptera) (Robertson et al. 2015). Within this superfamily, Coccinellidae is the most species‐rich family and contains tribes that differ widely in feeding ecology and morphology. Among these tribes, Coccinellini is taxonomically important and ecologically diverse because of its predominance of predatory species and its widespread use in biological control, motivating a focused phylogenomic investigation. The family Coccinellidae was traditionally classified into six or seven subfamilies (i.e., Chilocorinae, Coccidulinae, Coccinellinae, Epilachninae, Scymninae, Sticholotidinae, and sometimes Ortaliinae) (Hodek and Honěk 1996; Kovář 1996). This classification system was developed by Sasaji (1968, 1971) on the basis of comparative morphological analyses of adult and larval specimens from the Palaearctic species, with a focus on Japanese taxa. Kovář (1996) extended this to a global classification, recognizing seven subfamilies and 38 tribes. Ślipiński (2007) showed that those schemes do not form monophyletic groups and proposed a basal split of Coccinellidae into Microweiseinae and Coccinellinae. More recently, Che et al. (2021) further revised this system by dividing it into three subfamilies: Coccinellinae, Monocoryninae, and Microweiseinae, with Coccinellinae containing most of the tribes, including Coccinellini.

The tribe Coccinellini, first established by Latreille (Latreille 1807) with the genus *Coccinella* Linnaeus, 1758 as its type genus. It is one of the large and specious tribes in the family Coccinellidae, commonly referred to as ‘true ladybirds’, which comprises 90 genera and more than 1000 species worldwide (Escalona et al. 2017; Kovář 2007; Poorani 2002; Ślipiński 2007; Ślipiński and Tomaszewska 2010; Vandenberg 2002). This tribe exhibits distinct morphological diagnostic characters (Seago et al. 2011; Ślipiński and Tomaszewska 2010): adult females possess ovipositor‐associated glands of unknown function; larvae are highly mobile, with elongated front legs, aposematic, lacking paired dorsal glandular openings, and with a dorsal armature; pupae have a distinct sclerotized abdominal terga (I–VII) forming a gin trap‐like mechanism. Although most Coccinellini species are primarily aphidophagous, this tribe exhibits diverse feeding habits, with significant host shifts occurring throughout its evolutionary diversification (Escalona et al. 2017; Giorgi et al. 2009; Magro et al. 2010). Notable species within Coccinellini include *Coccinella septempunctata* Linnaeus (the seven‐spotted ladybird beetle) and widely used biocontrol agents such as *Adalia bipunctata* (Linnaeus) and *Hippodamia variegata* (Goeze) (Nattier et al. 2021).

The monophyly of Coccinellini has been well demonstrated by recent taxonomic revisions (Seago et al. 2011) and corroborated by molecular analyses conducted by Nattier et al. (2021) and Tomaszewska et al. (2021). What is now recognized as a single tribe was previously treated as five separate tribes: Tytthaspidini, Halyziini, Singhikaliini, Discotomini, and Coccinellini (Gordon 1985; Kovář 1996; Nedvěd and Kovář 2012; Sasaji 1968, 1971; Vandenberg 2002). More recent phylogenetic studies, utilizing both morphological (Seago et al. 2011) and molecular (Escalona et al. 2017) data, have transferred Discotomini, Halyziini (syn. Psylloborini), Singhikaliini, and Tytthaspidini (syn. Bulaeini) into Coccinellini. The evolutionary history of Coccinellini traces back to the Cretaceous period, approximately 105 Ma, coinciding with angiosperm diversification (McKenna et al. 2015). However, no definitive Cretaceous fossil records of Coccinellidae have been identified to date. The oldest confirmed Coccinellidae fossils originate from the Lower Eocene French Oise amber (ca. 53 Ma) (Kirejtshuk and Nel 2012) and the Middle Eocene Baltic amber (ca. 44 Ma) (Szawaryn 2019; Szawaryn and Szwedo 2018; Szawaryn and Tomaszewska 2020), highlighting a substantial gap between molecular divergence estimates and the fossil record.

In the present study, we obtained nine complete or nearly complete mitogenomes of ladybird species: *Coelophora circumvelata* (Mulsant 1850), *Harmonia dimidiata* (Fabricius 1781), *Harmonia yedoensis* (Takizawa 1917), *Maroilleis hauseri* (Mader 1930), *Micraspis satoi* Miyatake 1977, *Micraspis allardi* (Mulsant 1866), *Propylea luteopustulata* (Mulsant 1850), *Singhikalia duodecimguttata* Xiao and Li 1992 and *Synonia consanguihae* Poorani et al. 2008, using high‐throughput sequencing technology. In this study, we aim to (i) characterize mitogenome features of nine species; (ii) test monophyly and internal phylogenetic relationships of Coccinellini with expanded sampling by integrating 58 publicly available Coccinellidae mitogenomes from GenBank; (iii) estimate divergence times to place the evolutionary diversification of the tribe.

## 2 Materials And Methods

### 2.1 Taxon Sampling and DNA Extraction

Adult specimens of *Coelophora circumvelata, Harmonia dimidiata*, *H. yedoensis*, *Maroilleis hauseri*, *Micraspis allardi*, *M. satoi, Propylea luteopustulata, Singhikalia duodecimguttata*, and *Synonia consanguihae* were collected from China (Table 1) and preserved in 95% ethanol and at −20°C before DNA extraction. Species were initially identified on the basis of morphological characters provided by Pang and Gordon (1986), Canepari (2003), and Wang and Chen (2022). Identifications of *H. dimidiata* , *H. yedoensis*, and *M. allardi* were further confirmed using nucleotide BLASTN software (v2.13.0) (Chen et al. 2015) searches against NCBI reference sequences MT994285, PV204067, and OP263126, respectively. Total genomic DNA was extracted from the head and prothorax of the specimens using a DNeasy Blood and Tissue kit (TIANGEN, Beijing, China), eluted in 150 μL TE buffer, and stored at −80°C until further utilization. Voucher samples of the nine species were deposited at the Anhui Provincial Key Laboratory of the Conservation and Exploitation of Biological Resources, College of Life Sciences, Anhui Normal University. Photographic images were captured using a Canon EOS 850 with a 100 mm lens, and the photographs were edited using Helicon Focus v.8.1 and Adobe Photoshop 2024.

**TABLE 1:** List of reference mitochondrial genomes chosen for phylogenetic analysis.

Subfamily	Tribe	Species	Length (bp)	Acc. No.	References
Coccinellinae	Aspidimerini	*Cryptolaemus montrouzieri*	17,010	KT874575	Unpublished
Coccinellinae	Chilocorini	*Chilocorus bipustulatus*	1229	MN053054	Song et al. (2020)
Coccinellinae	Chilocorini	*Chilocorus rubidus*	16,801	OQ130027	Unpublished
Coccinellinae	Coccidulini	*Coccidula rufa*	10,589	JX412767	Unpublished
Coccinellinae	Coccinellini	*Adalia bipunctata*	18,463	MW029465	Unpublished
Coccinellinae	Coccinellini	*Adalia decempunctata*	19,684	OX637707	Schoch et al. (2020)
Coccinellinae	Coccinellini	*Aiolocaria hexaspilota*	17,549	MK583344	Seo et al. (2019)
Coccinellinae	Coccinellini	*Anatis ocellata*	17,092	KX035143	Unpublished
Coccinellinae	Coccinellini	*Anisosticta novemdecimpunctata*	15,289	KT876880	Unpublished
Coccinellinae	Coccinellini	*Aphidecta obliterata*	17,945	OZ014437	Schoch et al. (2020)
Coccinellinae	Coccinellini	*Calvia championorum*	17,575	KX132085	Unpublished
Coccinellinae	Coccinellini	*Calvia decemguttata*	16,425	KX087252	Unpublished
Coccinellinae	Coccinellini	*Calvia muiri*	17,575	MF992928	Song et al. (2020)
Coccinellinae	Coccinellini	*Cheilomenes sexmaculata*	17,297	MW845811	Cheng et al. (2021)
Coccinellinae	Coccinellini	*Coccinella lama*	18,932	MW029464	Li et al. (2021)
Coccinellinae	Coccinellini	*Coccinella septempunctata*	19,413	OU015583	Unpublished
Coccinellinae	Coccinellini	*Coccinella transversoguttata*	17,575	OK624419	Unpublished
Coccinellinae	Coccinellini	***Coelophora circumvelata***	**18,596**	**PX067362**	**this study**
Coccinellinae	Coccinellini	*Coelophora saucia*	14,106	MK574678	Zhou et al. (2019)
Coccinellinae	Coccinellini	*Coleomegilla maculata*	17,516	KJ778881	Paula et al. (2016)
Coccinellinae	Coccinellini	*Cycloneda munda*	14,292	KJ778882	Paula et al. (2016)
Coccinellinae	Coccinellini	*Cycloneda sanguinea*	15,118	KJ778883	Paula et al. (2016)
Coccinellinae	Coccinellini	*Eriopis connexa*	17,652	MG253268	Unpublished
Coccinellinae	Coccinellini	*Eriopis patagonia*	15,720	MN509443	Salazar and Nattier (2020)
Coccinellinae	Coccinellini	*Halyzia sedecimguttata*	15,766	KT780652	Unpublished
Coccinellinae	Coccinellini	*Harmonia axyridis*	16,387	KR108208	Niu et al. (2016)
Coccinellinae	Coccinellini	*Harmonia eucharis*	17,441	MW029462	Li et al. (2021)
Coccinellinae	Coccinellini	*Harmonia quadripunctata*	18,051	KX087296	Unpublished
Coccinellinae	Coccinellini	***Harmonia dimidiata***	**16,435**	**PX067370**	**this study**
Coccinellinae	Coccinellini	***Harmonia yedoensis***	**17,356**	**PX067368**	**this study**
Coccinellinae	Coccinellini	*Hippodamia convergens*	18,419	KX755331	Unpublished
Coccinellinae	Coccinellini	*Hippodamia tredecimpunctata*	17,275	KJ778889	Paula et al. (2016)
Coccinellinae	Coccinellini	*Hippodamia undecimnotata*	15,587	KX087298	Unpublished
Coccinellinae	Coccinellini	*Hippodamia variegata*	17,823	MK334129	Hao et al. (2019)
Coccinellinae	Coccinellini	*Illeis bistigmosa*	17,840	MZ325765	Zhu et al. (2022)
Coccinellinae	Coccinellini	*Illeis cincta*	15,856	MF992929	Song et al. (2020)
Coccinellinae	Coccinellini	*Illeis koebelei*	17,054	OK012004	Unpublished
Coccinellinae	Coccinellini	***Maroilleis hauseri***	**16,943**	**PX067369**	**this study**
Coccinellinae	Coccinellini	*Megalocaria dilatata*	18,608	MZ983384	Unpublished
Coccinellinae	Coccinellini	***Micraspis allardi***	**17,173**	**PX067364**	**this study**
Coccinellinae	Coccinellini	***Micraspis satoi***	**17,488**	**PX067363**	**this study**
Coccinellinae	Coccinellini	*Myrrha octodecimguttata*	20,609	OY294066	Schoch et al. (2020)
Coccinellinae	Coccinellini	*Oenopia dracoguttata*	19,220	MW029467	Timmermans et al. (2015)
Coccinellinae	Coccinellini	*Oenopia formosana*	17,885	OR804096	Unpublished
Coccinellinae	Coccinellini	*Oenopia sauzeti*	17,630	MW530420	Unpublished
Coccinellinae	Coccinellini	*Olla v‐nigrum*	14,448	MZ303015	Unpublished
Coccinellinae	Coccinellini	*Propylea japonica*	15,027	KM244660	Tang et al. (2014)
Coccinellinae	Coccinellini	*Propylea quattuordecimpunctata*	17,471	MF992931	Unpublished
Coccinellinae	Coccinellini	*Propylea* sp.	15,915	KX132084	Unpublished
Coccinellinae	Coccinellini	***Propylea luteopustulata***	**14,776**	**PX067365**	**this study**
Coccinellinae	Coccinellini	*Psyllobora lenta*	14,462	MZ303017	Unpublished
Coccinellinae	Coccinellini	***Singhikalia duodecimguttata***	**19,843**	**PX067366**	**this study**
Coccinellinae	Coccinellini	***Synonia consanguihae***	**18,792**	**PX067367**	**this study**
Coccinellinae	Coccinellini	*Vibidia duodecimguttata*	19,627	MT114193	Yan et al. (2020)
Coccinellinae	Epilanchnini	*Epilachna admirabilis*	17,445	MN053053	Song et al. (2020)
Coccinellinae	Epilanchnini	*Henosepilachna pusillanima*	16,216	KJ131489	Behere et al. (2016)
Coccinellinae	Epilanchnini	*Henosepilachna vigintioctopunctata*	17,057	MG584727	Song et al. (2020)
Coccinellinae	Subcoccinellini	*Subcoccinella vigintiquattuorpunctata*	14,645	KT780695	Unpublished
Coccinellinae	Epivertini	*Epiverta chelonia*	17,347	ON209194	Zhang et al. (2023)
Coccinellinae	Hyperaspidini	*Brachiacantha groendali*	15,499	MZ303003	Unpublished
Coccinellinae	Hyperaspidini	*Hyperaspis festiva*	15,999	MZ303012	Unpublished
Coccinellinae	Hyperaspidini	*Thalassa montezumae*	16,976	PP865227	Iovinella et al. (2024)
Coccinellinae	Scymnini	*Nephus (Bipunctatus) includens*	16,638	MN164642	Magro et al. (2020)
Coccinellinae	Scymnini	*Nephus (Nephus) oblongosignatus*	16,647	MT445723	Magro et al. (2020)
Coccinellinae	Scymnini	*Scymnus (Pullus) canariensis*	17,646	OQ716382	Jiménez‐García et al. (2023)
Coccinellinae	Scymnini	*Scymnus (Pullus) cardi*	15,416	PP639204	Iqbal et al. (2024)
Microweiseinae	Microweiseini	*Coccidophilus cariba*	15,343	MN447521	Nattier and Salazar (2019)

### 2.2 Genome Sequencing, Mitogenome Assembly, and Annotation

Whole‐genome sequencing libraries were prepared from genomic DNA following standard Illumina paired‐end protocols, without mitochondrial DNA enrichment or targeted amplification. Each specimen was processed as an independent library and sequenced on the Illumina NovaSeq 6000 platform (Berry Genomics Corporation, Beijing, China), generating 150 bp paired‐end reads with an average insert size of approximately 350 bp to produce low‐coverage whole genome data. Mitochondrial genomes were subsequently assembled bioinformatically from these sequencing reads.

Initially, adapter trimming was conducted using Trimmomatic v0.36 (Bolger et al. 2014), followed by the removal of low‐quality and short reads using Prinseq v0.20.4 (Schmieder and Edwards 2011). Subsequently, high‐quality reads were de novo assembled with GetOrganelle v1.7.7.0 (Jin et al. 2020) employing K‐mer sizes of 21, 45, 65, 85, and 105, with a *t*‐value of 15 (Li et al. 2020; Yan et al. 2020; Yuan et al. 2020). Gene annotations, circularization verification, and extraction of individual protein‐coding genes and rRNAs from the mitochondrial genome were carried out using Geneious Prime v2025.0.2 (Kearse et al. 2012). The mapping of mitogenomes was conducted using the CGView Server (Grant and Stothard 2008). AT‐skew and GC‐skew were calculated using the formulas: AT‐skew = [A%−T%] / [A% + T%] and GC‐skew = [G%−C%] / [G% + C%] (Perna and Kocher 1995). Codon usage and relative synonymous codon usage (RSCU) for the 13 PCGs were determined using PhyloSuite v1.2.3 (Xiang et al. 2023; Zhang et al. 2020). The nonsynonymous (Ka) to synonymous (Ks) substitution ratio for the 13 PCGs was calculated with DnaSP v6.0 (Barcelona, Spain) (Rozas et al. 2017). Transfer RNA (tRNA) genes and their secondary structures were annotated using the MITOS pipeline implemented via the Galaxy platform (Bernt et al. 2013), which was used to predict tRNA secondary structures and identify anticodons in the mitochondrial genomes. Substitution saturation tests were performed on three datasets (13 PCGs_codon1, 13 PCGs_codon2, 13 PCGs_codon3) using DAMBE v7 (Xia 2018) with the GTR model (Perna and Kocher 1995).

### 2.3 Phylogenetic Analyses

We analyzed mitochondrial genome sequences from 67 species (50 species of Coccinellini and 17 species from related tribes) to investigate the phylogenetic relationships of the tribe at different taxonomic levels and confirm the systematic positions of nine target species (*Coelophora circumvelata, Harmonia dimidiata*, *H. yedoensis*, *Maroilleis hauseri*, *Micraspis allardi*, *M. satoi, Propylea luteopustulata, Singhikalia duodecimguttata*, and *Synonia consanguihae*) (Table 1). Phylogenetic analyses were conducted using two datasets: (i) the nucleotide sequences of the 13 mitochondrial protein‐coding genes (PCGs_NT) and (ii) the corresponding amino acid sequences (PCGs_AA). The two datasets were aligned separately using MAFFT v7.313 (Katoh and Standley 2013), with codon structure explicitly considered for nucleotide alignments. For the nucleotide sequence alignments, poorly aligned regions, gaps, and ambiguous sites were removed with Gblocks v0.91b (Talavera and Castresana 2007) under default parameters. The amino acid alignments were filtered using trimAl v1.2 (Capella‐Gutiérrez et al. 2009) with the “‐automated1” option. For improved alignment accuracy, nucleotide sequences of PCGs were further refined using MACSE v2.03 (Ranwez et al. 2018). The aligned gene datasets were then concatenated in PhyloSuite (Xiang et al. 2023; Yuan et al. 2020) for downstream phylogenetic reconstruction.

The phylogenetic trees of both datasets were constructed using Bayesian inference (BI) and maximum likelihood (ML) methods. Bayesian inference analysis was performed using PhyloBayes MPI v1.5a (Lartillot et al. 2013) under the CAT‐GTR model. Two independent Markov chain Monte Carlo runs were performed until convergence was reached (maximum discrepancy < 0.1). Following a burn‐in of the first 25% of sampled trees, a majority‐rule consensus tree was generated from the combined post‐burn‐in trees of both runs. Maximum likelihood phylogenetic tree construction employed IQ‐TREE v2.2.0 (Minh et al. 2020), with the MFP‐MERGE to select best‐fit models and the final partition scheme. Node support in all maximum likelihood analyses was determined through 1000 SH‐aLRT repeats (Guindon et al. 2010) and 1000 UFBoot2 bootstraps (‐B 1000, ‐alrt 1000) (Hoang et al. 2018).

### 2.4 Divergence Time Estimation

Divergence times were estimated using PCGs_AA dataset in the BEAST v.1.10.4 (Suchard et al. 2018), with the Bayesian MCMC method under an uncorrelated lognormal relaxed clock model to calculate rate heterogeneity and the Yule prior method for the tree prior. The input file for BEAST was generated using a program BEAUti v.1.10.4 (distributed with BEAST) (Suchard et al. 2018). For this study, we adopted a node calibration approach on the basis of primary fossil data from the literature (Cockerell 1906; Förster 1891; Kirejtshuk and Nel 2012) and applied it at four nodes corresponding to the genera *Coccinella*, *Chilocorus*, *Nephus*, and *Scymnus* (Table S1).

To prevent changes in tree topology, we used the PhyloBayes tree (PCGs_AA dataset) as the starting tree and disabled the narrow and wide exchange, Wilson balding operators, and subtree sliding in BEAST. We performed four independent MCMC runs of 50 million generations, sampling every 5000 steps. The convergence and effective sample sizes (ESS) of parameters were assessed using Tracer v1.6 (Rambaut 2000). To determine the best‐fitting model, four runs of clock model were tested. The resulting tree files from each run were combined by LogCombiner v1.10.4 (Suchard et al. 2018), and a final Maximum Clade Credibility (MCC) tree was generated with TreeAnnotator v1.10.4 (Suchard et al. 2018), discarding 20% of the initial trees as burn‐in.

## 3 Results

### 3.1 The Structure of the Mitochondrial Genomes

The newly obtained mitogenomes were sequenced to a total output of approximately 6 gigabases (Gb) using high‐throughput sequencing technology.

The mitochondrial genome sizes ranged from 14,776 base pairs (bp) in *Propylea luteopustulata* to 19,843 bp in *Singhikalia duodecimguttata*, with notable variations primarily observed in the control region (Tables S2–S10).

**FIGURE 1:**
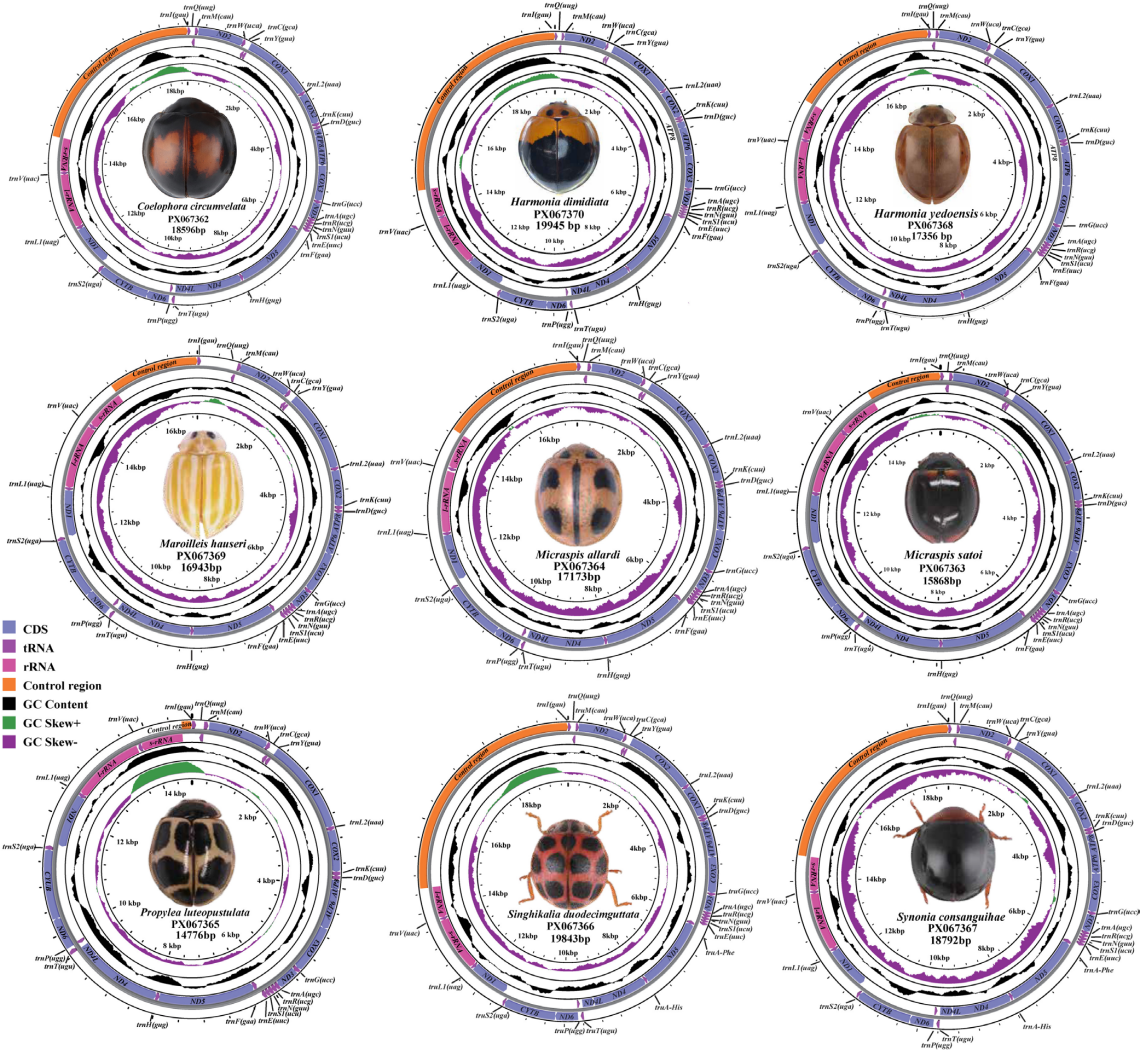
Circular diagram of the nine newly sequenced mitogenomes of Coccinellini.

All nine new mitochondrial genomes of Coccinellini species: *Coelophora circumvelata, Harmonia dimidiata*, *H. yedoensis*, *Maroilleis hauseri*, *Micraspis allardi*, *M. satoi, Propylea luteopustulata*, *Singhikalia duodecimguttata*, and *Synonia consanguihae*, comprise 37 genes, including 13 protein‐coding genes, 2 ribosomal RNA genes, 22 transfer RNA genes, and a substantial non‐coding region (Control Region). Among them, nine protein‐coding genes and 14 transfer RNA genes are situated on the positive strand (J strand), whereas the remaining four protein‐coding genes, eight transfer RNA genes, and two ribosomal RNA genes are located on the negative strand (N strand) (Figure 1). The gene arrangement of all newly sequenced mitogenomes is similar to that of the other previously studied Coccinellidae mitogenomes (Behere et al. 2016; Cheng et al. 2021; Hao et al. 2019; Iovinella et al. 2024; Iqbal et al. 2024; Kim et al. 2012; Li et al. 2021; Magro et al. 2020; Niu et al. 2016; Salazar and Nattier 2020; Song et al. 2020; Yan et al. 2020; Zhang et al. 2023; Zhou et al. 2019; Zhu et al. 2022) and possesses the complete set of mitochondrial genes characteristic of insect mitogenomes.

The mitogenomes of the nine newly sequenced species exhibited a strong A + T bias, with overall A + T content averaging 78.2% and ranging from 74.9% in *Harmonia dimidiata* to 80.1% in *Synonycha consanguinea*. A + T composition was highest in the control region (83.5%), followed by rRNAs (80.3%), tRNAs (79.8%), and protein‐coding genes (77.7%) (Table S2).

The total length of the 13 protein‐coding genes (PCGs) ranged from 9414 bp in *Singhikalia duodecimguttata* to 11,055 bp in *Coelophora circumvelata*, with A + T content varying from 74.7% to 80.4% (Tables S3–S11). All PCGs initiated with the typical ATN start codon except cox1 in *C. circumvelata*, which used TCG. Complete stop codons (TAA or TAG) were present in 6–9 PCGs depending on species, whereas incomplete stop09;+ T bias, with overall A + T content averaging 78.2% and ranging from 74.9% in *Harmonia dimidiata* to 80.1% in *Synonycha consanguinea*. A + T composition was highest in the control region (83.5%), followed by rRNAs (80.3%), tRNAs (79.8%), and protein‐coding genes (77.7%) (Table S2).

The total length of the 13 protein‐coding genes (PCGs) ranged from 9414 bp in *Singhikalia duodecimguttata* to 11,055 bp in *Coelophora circumvelata*, with A + T content varying from 74.7% to 80.4% (Tables S3–S11). All PCGs initiated with the typical ATN start codon except cox1 in *C. circumvelata*, which used TCG. Complete stop codons (TAA or TAG) were present in 6–9 PCGs depending on species, whereas incomplete stop codons (T or TA) were frequently observed in atp6, cox1–3, nad1, nad2, nad4, nad5, and nad6 (Tables S3–S11). Codon usage showed a strong preference for A/T‐rich codons, with UUA, UCU, and CCU most frequent, reflecting the high A + T bias; trnL2, trnI, trnP, and trnM were the most frequently encoded amino acids (Figure S1). The rRNA genes showed conserved structure, with rrnL (1263–1344 bp) located between trnL2 (TAG) and trnV (TAC), and rrnS (697–816 bp) located between trnV and the control region. The 22 tRNAs generally exhibited conserved cloverleaf secondary structures, except for trnS1 (anticodon TCT), which lacked the DHU arm, and trnP, which lacked the TΨC arm, forming simple loops (Figures S2–S4), as reported in other ladybird and metazoan mitogenomes (Huang et al. 2023).

### 3.2 Evolution Rate and Substitution Saturation Analysis

**FIGURE 2:**
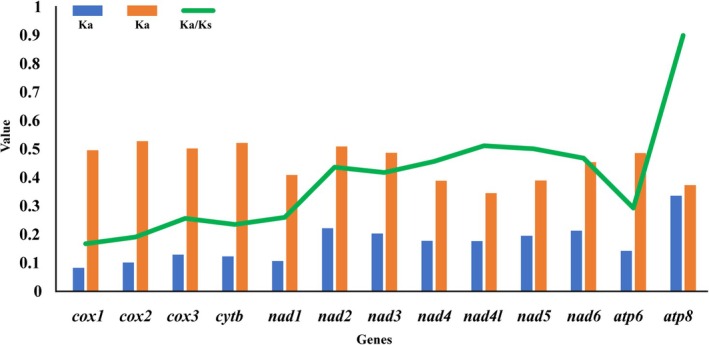
Average evolutionary rate among mitochondrial PCGs of nine newly sequenced species.

The average ratio of nonsynonymous substitution rate (Ka) to synonymous substitution rate (Ks) was computed for 13 protein‐coding genes across 67 species of ladybird beetles. The results revealed that the Ka/Ks substitution ratios for all 13 protein‐coding genes were below 1 (Figure 2). These findings indicate that all protein‐coding genes underwent purifying selection. The ranking of evolutionary rates among the 13 protein‐coding genes was as follows: *atp8* > *nad4l* > *nad5* > *nad6* > *nad4* > *nad2* > *nad3* > *atp6* > *nad1* > *cox3* > *cytb* > *cox2* > *cox1*. Notably, *cox1* exhibited the slowest evolutionary rate, whereas *atp*8 and *nad*6 displayed accelerated evolutionary rates and greater diversity compared to the other protein‐coding genes (Figure 2).

**FIGURE 3:**
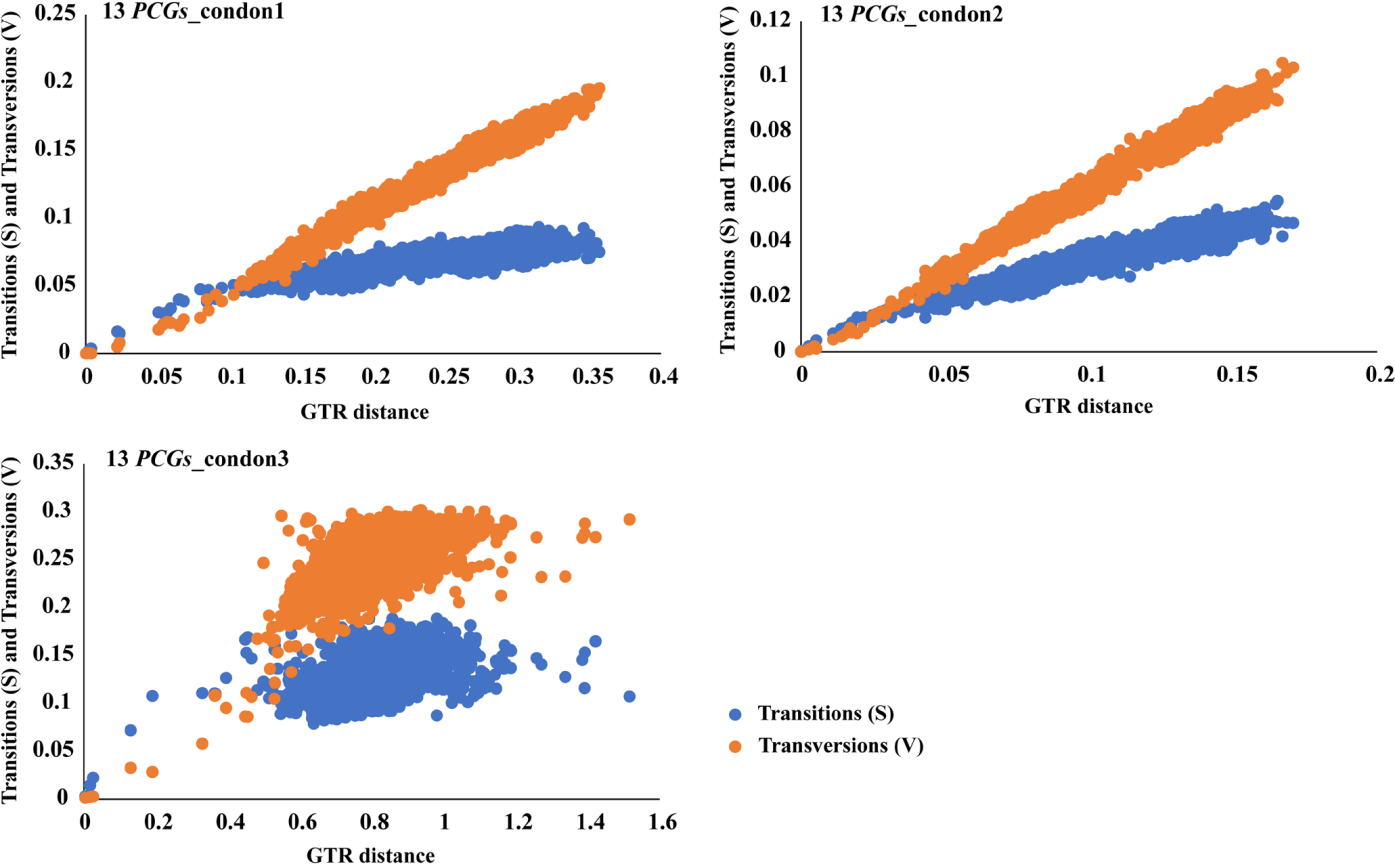
Saturation analysis on all three codon positions (1st, 2nd, and 3rd) across 13PCGs.

Prior to constructing the phylogenetic tree, we conducted substitution saturation analysis on the first, second, and third codons of 13 protein‐coding genes (13PCGs_codon1, 13PCGs_codon2, and 13PCGs_codon3) using DAMBE v.7. The analysis revealed that ISS values (substitution saturation simple index) for codon1, codon2, and codon3 were below the critical ISS values (ISS.c) (*p* < 0.05) (Figure 3). Additionally, there was a strong linear correlation observed between GTR distance and uncorrected pairwise differences in transitions and transversions. Notably, codon3 exhibited a marginal tendency towards saturation on the graph; however, the ISS value remained lower than the critical ISS value (ISS.c) (*p* < 0.05) (Figure 3). In summary, all three codon positions contain adequate phylogenetic information for the construction of the phylogenetic tree.

### 3.3 Phylogenetic Relationships

**FIGURE 4:**
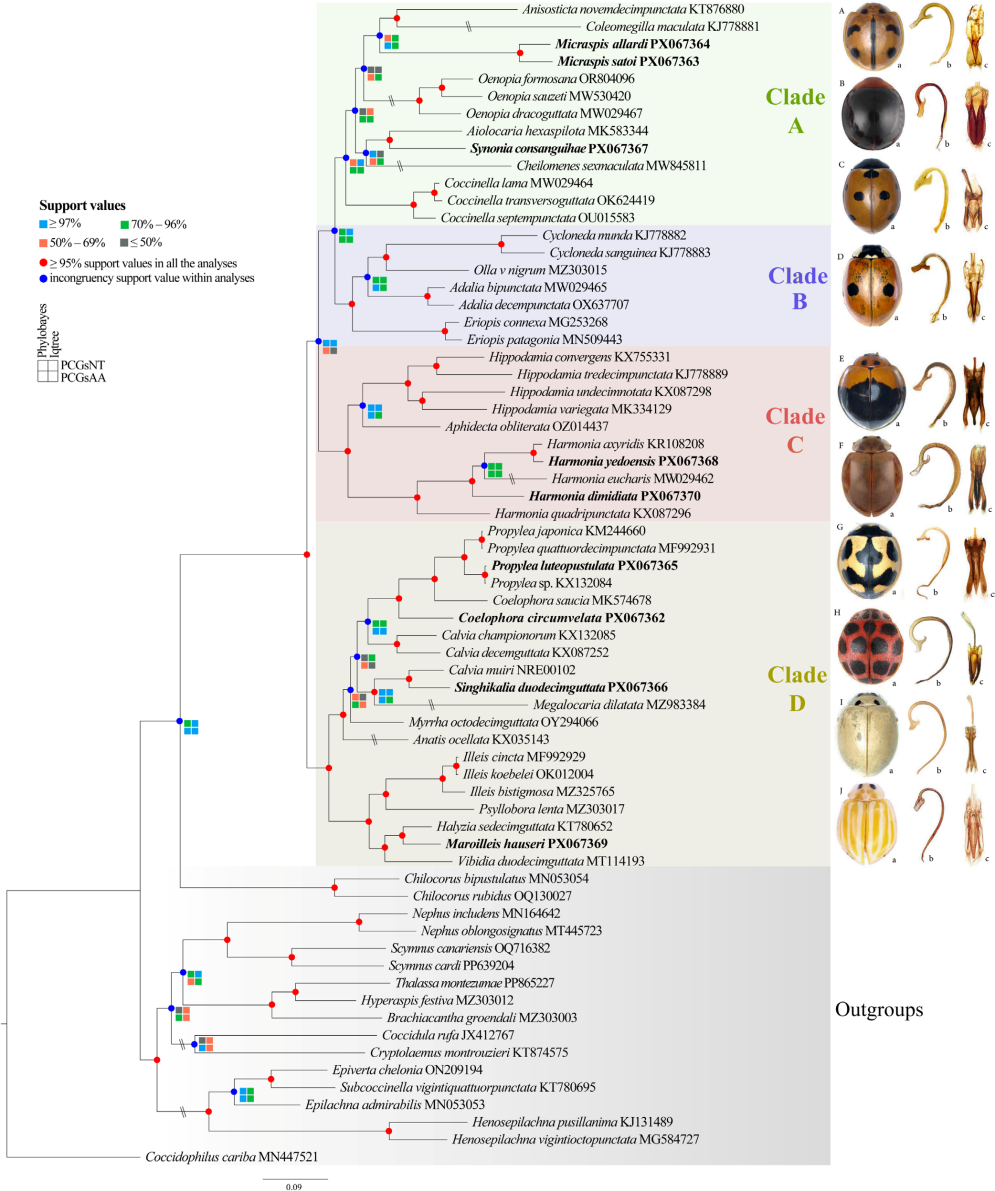
Phylogeny of Coccinellini using Bayesian inference (BI) on the basis of amino acid sequences of protein‐coding genes (PCGs_AA dataset). The bootstrap values of maximum‐likelihood analyses and posterior probabilities of Bayesian analyses are summarized and labeled around each node. Newly sequenced species are marked in bold. Conflicting topological positions between trees are denoted by double slashes (\). The phylogenetic tree illustrates specimens of the following taxa: (A) *Micraspis allardi* *;* (B) *Synonia consanguihae*; (C) *Coccinella septempunctata*; (D) *Adalia bipunctata*; (E) *Harmonia dimidiata*; (F) *Harmonia yedoensis*; (G) *Propylea luteopustulata*; (H) *Singhikalia duodecimguttata*; (I) *Illeis koebelei*; (J) *Maroilleis hauseri*. Elements of Morphology: (A) adult; (B) penis; (C) inner view of tegmen.

Phylogenetic analyses were reconstructed using both Maximum likelihood (ML) and Bayesian inference (BI) methods on two different datasets (PCGs_NT and PCGs_AA). The topology was largely consistent across all analyses, with most nodes exhibiting robust support (≥ 97%), as indicated by the color‐coded support values on the tree (Figure 4). However, the support for the ML tree was higher than that of the BI tree (Figure 4 and Figures S5–S8).

The phylogenetic results showed that the species of tribe Coccinellini were robustly inferred as the sister group to Chilocorini (BS = 99%, PP = 1.0), and the monophyly of Coccinellini was strongly confirmed (BS = 100%, PP = 1.0). The nine newly sequenced species: *Coelophora circumvelata, Harmonia dimidiata*, *H. yedoensis*, *Maroilleis hauseri, Micraspis satoi*, *Micraspis allardi*, *Propylea luteopustulata*, *Singhikalia duodecimguttata*, and *Synonia consanguihae*—were clustered within the Coccinellini clade, confirming their taxonomic placement.

The main clade of tribe Coccinellini is divided into four distinct clades (A—D) with high support values in all phylogenetic analyses. Across all analyzed datasets and both Maximum Likelihood and Bayesian Inference methods, these four clades were consistently recovered as monophyletic with the following relationship: (Clade D, (Clade C, (Clade B, Clade A))).

‘Clade A’ comprises eight genera. The cosmopolitan genus *Micraspis* emerges as the sister group to *Coleomegilla* + *Anisosticta*. *Micraspis satoi* and *Micraspis allardi* form a highly supported monophyletic group (BS ≥ 70%, PP ≥ 0.97). *Synonia consanguihae* is strongly supported as sister to *Aiolocaria* (BS ≥ 70%, PP ≥ 0.97), whereas *Coccinella* is in the basal position of this clade. Morphologically, these genera exhibit the following similar diagnostic characters: the head is partially concealed under the pronotum but dorsally exposed; the pronotum is broad; in many species, the anterior margin of the pronotum is straight or slightly curved. However, each genus has distinct morphological character; for example, male genital characters (penis and tegmen) are useful for genus or species level identification (see Figure 4).

‘Clade B’, with strong statistical support (BS ≥ 70%, PP ≥ 0.97) in all the analyses, includes the species from *Adalia*, *Cycloneda*, *Eriopis*, and *Olla*. All these genera share conspicuous pronotal ornamentation patterns, such as the M‐ or V‐shaped marking in *Adalia*, *Cycloneda*, and *Olla*, whereas *Eriopis* shows black pronotum markings with white or pale anterolateral margins. As with Clade A, male genital architecture provides critical taxonomic resolution at both generic and species levels (Figure 4).

‘Clade C’ (containing *Aphidecta*, *Harmonia*, and *Hippodamia*) was strongly supported in most analyses (BS ≥ 70%, PP ≥ 0.97), except in PCGs_AA (BI) dataset (PP = 0.53). Within this clade, monotypic *Aphidecta* is sister to *Hippodamia*, whereas *Harmonia* forms a monophyletic group, including the closely related *H. dimidiata* and *H. yedoensis* (BS ≥ 70%, PP ≥ 0.97) (Figure 4).

‘Clade D’, consisting of 12 genera, was also strongly supported as monophyletic in all analyses (BS ≥ 70%, PP ≥ 0.97). The genus *Calvia*, *Coelophora*, and *Propylea* cluster as to form a subclade, whereas *Calvia muiri* is sister to *Singhikalia duodecimguttata*, with strong support (BS = 100%, PP = 0.97). The genera *Anatis*, *Myrrha*, and *Megalocaria* were recovered as a monophyletic group with weak support (BS > 50%, PP > 0.70) in all analyses. Similarly, the mycophagous species grouped together in the same subclade with high support (BS = 100%, PP = 1), consisting of *Halyzia*, *Illeis*, *Psyllobora*, and *Vibidia*, which are also recovered as monophyletic. All these 12 genera are taxonomically distinct (Figure 4) but are shown to be closely related on the basis of their mitochondrial genomes.

Phylogenetic analyses revealed conflicting topologies regarding the relationships of certain genera across different datasets and methods. *Anisosticta* showed inconsistency, grouping either with *Coleomegilla* in PCGs_NT (BI) (Figure S6) but with the *Micraspis* in PCGs_AA (ML) (Figure S7). Similarly, in PCGs_NT (BI), *Cheilomenes* grouped with *Synonycha* and *Aiolocaria*, whereas *Coleomegilla* was sister *to Coccinella* species (Figure S6). In contrast, PCGs_AA (ML) recovered *Cheilomenes* with *Anisosticta* and *Coleomegilla*, and the *Micraspis* genus remained as a distinct clade (Figure S7). Within Clade C, the grouping of *Anatis*, *Myrrha*, *Megalocaria*, *Singhikalia*, and *Calvia* is controversial across all phylogenetic analyses (Figure 4 and Figures S5–S8). The genus *Harmonia* also exhibited inconsistent relationships: *H. dimidiata* and *H. eucharis* formed a sister pair in PCGs_NT (BI) but appeared separately in other datasets (Figure S6). Among outgroups, Aspidimerini (*Cryptolaemus*), Coccidulini (*Coccidula*), and Hyperaspidini showed different associations depending on the dataset: either forming a sister group among themselves or grouping with Scymnini (Figure 4 and Figures S5–S8).

### 3.4 Divergence Time Estimation

**FIGURE 5:**
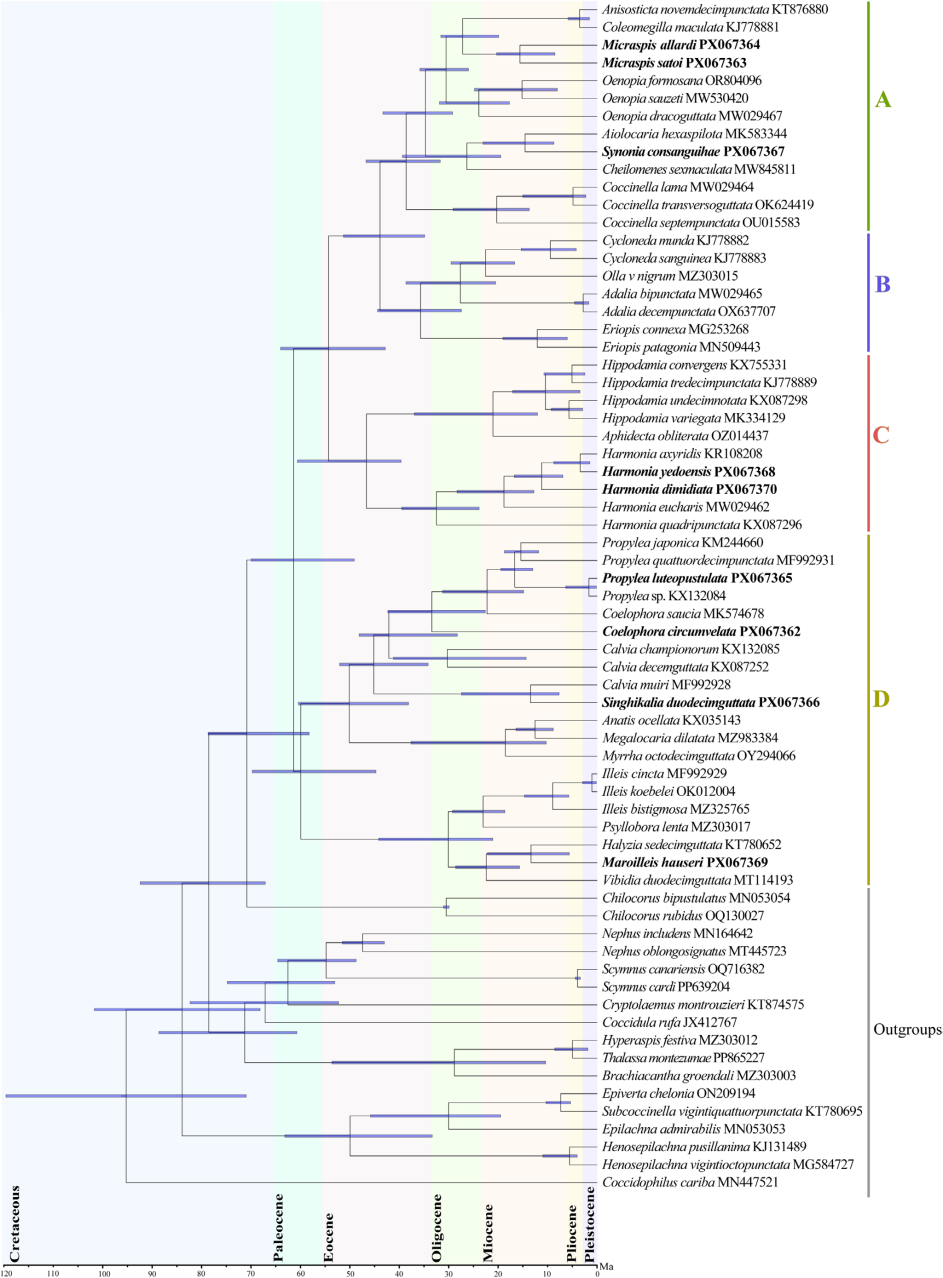
Divergence time estimation tree of Coccinellidae with emphasis on tribe Coccinellini using a Yule model in BEAST. The blue node bars show the ranges of the time estimates.

The molecular dating analysis used fossil‐calibrated crown nodes (Table S1) under an uncorrelated lognormal relaxed clock with a Yule tree prior. Our divergence time estimates indicate that the family Coccinellidae originated approximately 120 million years ago (Ma) (95% highest posterior density [HPD]: 95–124 Ma) (Figure 5). Within Coccinellidae, the crown group of the tribe Coccinellini was inferred to have emerged around 74 Ma, placing its origin in the Late Cretaceous. Subsequently, the four major Coccinellini clades (A–D) diversified in the early Paleogene: clade A at 43.1 Ma (95% HPD: 36.0–55.6 Ma), clade B at 35.0 Ma (95% HPD: 27.3–43.4 Ma), clade C at 45.0 Ma (95% HPD: 38.2–62.5 Ma), and clade D at 59.0 Ma (95% HPD: 44.1–70.0 Ma). Notably, the major lineages of Coccinellini began to diverge shortly before the Cretaceous–Paleogene (K–Pg) boundary (ca. 66 Ma) (Figure 5).

## 4 Discussion

### 4.1 Phylogenetic Relationship

Phylogenetic analyses robustly placed all nine newly sequenced species within the Coccinellini clade, corroborating their current taxonomic classification. The monophyly of Coccinellini is strongly supported (BS ≥ 70%, PP ≥ 0.97), and this clade is nested within the Coccinellinae. We recovered Coccinellini as sister to tribe Chilocorini, which is consistent with previous phylogenetic studies of the family Coccinellidae (Che et al. 2017; Escalona et al. 2017; Iqbal et al. 2024; Li et al. 2020; Magro et al. 2010; Nattier et al. 2021; Seago et al. 2011; Tomaszewska et al. 2021). Recently, Che et al. (2021), conducted a phylogenetic analysis of family Coccinellidae on the basis of extensive sampling and revealed that Coccinellini and Sticholotidini form a sister group. Therefore, adequate sampling of nuclear makers and whole genomes is needed to resolve the relationship of this tribe.

Mycophagous genera such as *Halyzia*, *Vibidia*, *Psyllobora*, and *Illeis* were also recovered within Coccinellini, in agreement with previous studies (Escalona et al. 2017; Iqbal et al. 2024; Magro et al. 2010; Nattier et al. 2021; Tomaszewska et al. 2021). Almost all the genera of Coccinellidae were also recovered as monophyletic (Figure 4 and Figures S5–S8). In the phylogenetic analyses by Nattier et al. (2021) and Tomaszewska et al. (2021), the tribe Coccinellini was divided into four main clades, but the relationships among these clades were inconsistent because of different datasets and species sampling. Our results support the phylogenetic relationship (Clade D, (Clade C, (Clade B, Clade A))), as also recovered by Nattier et al. (2021). However, Tomaszewska et al. (2021) proposed a different relationship (((Clade D, Clade C), Clade B), Clade A) on the basis of a combined matrix of morphology plus five nuclear loci (CAD × 2, TOPO, WGL, 3059fin) and COI for 164 species in 57 genera. This conflict likely reflects differences in marker composition, taxon sampling, and modeling; notably, the site‐heterogeneous CAT‐GTR model reduces compositional bias and reinforces the (B, A) sister relationship in our results.

The monophyly of ‘Clade A’ received strong support exclusively under Maximum Likelihood (BS ≥ 90%), a finding consistent with the phylogenetic framework proposed by Nattier et al. (2021), especially regarding the placement of *Coccinella* as sister to the remaining genera of the clade (Escalona et al. 2017). The grouping of *Aiolocaria* and *Synona* was also recovered by Escalona et al. (2017), Nattier et al. (2021), and Tomaszewska et al. (2021). Interestingly, the grouping of *Cheilomenes* with *Anisosticta*, *Coleomegilla*, and *Micraspis* in dataset PCGs_AA (ML) (weak supporting value; BS = 49) differs from other studies (Escalona et al. 2017; Magro et al. 2010; Nattier et al. 2021; Tomaszewska et al. 2021), which may be due to model effects in this dataset (as the selected models were mtART+*R*, mtART+I + G, mtMet+F + I + G, and mtZOA+F + I + I + *R*), rather than a robust historical pattern. However, these genera share a series of morphological characters: labial apical palpomere distinctly shorter than the penultimate one and the penis consists of more than one sclerite and spermatheca with a developed ramus and without a nodulus. Further morphological and phylogenetic studies are needed using a comprehensive sampling to revise these genera.

In ‘Clade B’, the relationship inferred between *Adalia*, *Cycloneda*, *Eriopis*, and *Olla*, was also recovered in previous studies (Escalona et al. 2017; Magro et al. 2010; Nattier et al. 2021; Tomaszewska et al. 2021). *Adalia* is widely distributed throughout the Oriental, Palearctic, Nearctic, and Neotropical regions (Waterhouse and Sands 2001), and consists of approximately 35 species (Gordon 1985). However, *Adalia* species of the Palearctic and Neotropical regions exhibit differences in male genitalia (Bielawski 1984; Dode 2011; González et al. 2017): Palearctic species have straight parameres, a phalobase in tegmen wider than long, and the distal part of the penis is moderately widened, whereas in Neotropical species, the parameres are distinctly curved in the middle towards the base, the phalobase in tegmen is longer than wide, and the distal part of the penis is widened. This highlights the need for taxonomic clarification, especially since the last comprehensive review of the genus focused only on Palearctic species (Iablokoff‐Khnzorian 1982).

Similarly, the relationship of ‘Clade C’ is congruent with previous studies (Escalona et al. 2017; Giorgi et al. 2009; Magro et al. 2010; Song et al. 2020). In contrast to Nattier et al. (2021), our results recover the monotypic *Aphidecta* as sister to *Hippodamia*, with strong support. The relationships of most genera in ‘Clade D’ were also recovered in previous studies (Escalona et al. 2017; Magro et al. 2010; Nattier et al. 2021; Song et al. 2020; Tomaszewska et al. 2021). However, we were unable to clarify the relationships among species and genera, such as *Anatis*, *Megalocaria*, and *Myrrha*, because of a lack of large sampling data. Here, the paraphyly of *Calvia* is due to the position of *Calvia muiri*, which is sister to *Singhikalia duodecimguttata*. This genus was also recovered as paraphyletic in the study of Song et al. (2020) and polyphyletic in the studies of Nattier et al. (2021) and Tomaszewska et al. (2021). *Calvia* is widely distributed across the Palearctic region, including 20 species (Booth 1997; Iablokoff‐Khnzorian 1982; Poorani 2004, 2014), with only a single Nearctic species ( *C. quatuordecimguttata* ) (Gordon 1985). However, this genus still needs to be revised because of inconsistent sets of diagnostic characters used to describe Palearctic and Nearctic species (Poorani 2004).

### 4.2 Divergence Time Estimation

Recent molecular studies indicate that the superfamily Coccinelloidea, which includes family Coccinellidae (ladybird beetles), probably originated during the Jurassic period, approximately 160–180 Ma (McKenna et al. 2019, 2015; Zhang et al. 2018). The diversification of the ladybird beetle dates back to 100–150 Ma. Importantly, there are no confirmed fossil representatives of the tribe Coccinellini, complicating precise estimates of its divergence times. However, molecular dating analyses conducted by Nattier et al. (2021) and Tomaszewska et al. (2021) estimated divergence times within the tribe Coccinellini using a combination of fossil calibration and crown‐group constraints. Our analyses support an Early Cretaceous origin for crown‐group Coccinellidae, consistent with estimates from previous molecular dating studies (Nattier et al. 2021; Tomaszewska et al. 2021), and place the emergence of the Coccinellini crown‐group in the Late Cretaceous (74 Ma) (Figure 5). This timing is significant, as it closely precedes the Cretaceous–Paleogene (K–Pg) boundary, a period marked by major global environmental changes and mass extinction events (Schulte et al. 2010).

The rapid radiation of Coccinellini generic clades during the Paleogene–Neogene transition parallels contemporaneous diversification events observed in Sternorrhyncha (Hemiptera) (Li et al. 2017; Von Dohlen and Moran 2000), a major group of hemipteran insects that includes many aphid and scale insect lineages—prominent prey for coccinellids. The temporal overlap between Coccinellini and Sternorrhyncha radiations suggests that predator–prey dynamics, possibly mediated by the widespread and rapid expansion of angiosperms, were a major driver of adaptive diversification in both groups. The evolutionary diversification of Coccinellini was likely facilitated by novel ecological niches and expanded trophic resources resulting from angiosperm radiation during the Cretaceous‐Tertiary transition, as evidenced by (Tomaszewska et al. 2021). However, as with all fossil‐calibrated molecular dating, some uncertainty remains because of limitations in the fossil record and potential rate heterogeneity among lineages.

## 5 Conclusions

This study expands mitochondrial genomes resources for the tribe Coccinellini by presenting and characterizing nine newly sequenced mitogenomes and using them, together with available data, to reconstructs phylogenetic and evolutionary relationships within the tribe. The mitogenomic features were highly conserved across the sampled species, supporting their taxonomic placement within Coccinellini. Our analyses strongly support the monophyly of Coccinellini and reveal a consistent phylogenetic structure across all datasets and analytical methods. The recovered sister relationship between Coccinellini and Chilocorini corroborates prior molecular phylogenies, although conflicting with traditional morphological‐based classifications. Phylogenetic analyses robustly resolve Coccinellini into four major clades (Clades A–D). The internal phylogeny within Coccinellini revealed existing relationships among the genera, highlighting the complexity of their evolutionary history. The results of divergence time estimation indicated a close evolutionary link between ladybird beetle diversification and the ecological expansion of their prey particularly Sternorrhyncha on flowering plants. Future research should include sampling from a broader taxonomic group and nuclear genomic data to further refine the evolutionary history of this beetle group, which is significant in both ecology and agriculture.

## Author Contributions

**Xin‐Yi Li:** conceptualization (equal), formal analysis (equal), methodology (equal), software (equal), validation (equal), writing – original draft (equal). **Zafar Iqbal:** conceptualization (equal), data curation (lead), formal analysis (equal), methodology (equal), software (equal), validation (equal), visualization (equal), writing – original draft (equal). **Fang Qi:** formal analysis (equal), investigation (equal), software (equal), visualization (equal). **Lin Xiaoling:** formal analysis (equal), resources (equal), software (equal), validation (equal), visualization (equal). **Xing‐Min Wang:** conceptualization (equal), formal analysis (lead), investigation (equal), methodology (equal), supervision (equal), visualization (equal), writing – review and editing (equal). **Rui‐E Nie:** conceptualization (equal), data curation (supporting), funding acquisition (lead), investigation (equal), methodology (lead), project administration (lead), software (equal), supervision (lead), visualization (equal), writing – review and editing (lead).

## Funding

This work was supported by the National Science Foundation of China,32170443, 32570538. Anhui Provincial University Outstanding Youth Program, 2022AH020021. The Anhui Provincial University Innovation and Entrepreneurship Training Program, 20241370126.

## Conflicts of Interest

The authors declare no conflicts of interest.

## Supporting information


**Data S1:** Supporting Information.

**Data S2:** Supporting Information.

## Data Availability

The following information was supplied regarding the availability of DNA sequences: the new mitogenomes are deposited in GenBank (https://www.ncbi.nlm.nih.gov/genbank/) and the accession numbers are PX067362—PX067370.
