# Supplementary material for: Mitogenomic Characterization and Phylogenetic Expansion of Tribe Coccinellini (Coleoptera: Coccinellidae)
Source: Ecol Evol. 2026 Mar 12;16(3):e73241. doi: 10.1002/ece3.73241 (PMC13093454; doi:10.1002/ece3.73241)
Supplement: Supplementary file 2 — Data S2: Supporting Information. [file ECE3-16-e73241-s002.docx]

| **TABLE S1 \|** Fossil and reference information for dating analysis. | | | | | | | |
| --- | --- | --- | --- | --- | --- | --- | --- |
| **No.** | **Calibrated nodes** | **Fossils** | **Age Distribution (Ma)** | **Age estimate (Ma)** | **References** | **Locality** | **Time interval** |
| 1 | *Nephus* and related genera | *Nephus subcircularis* | 40 - 50 | 48.6 ± 1.5 | Kirejtshuk & Nel, 2012 | Oise amber, France | Ypresian |
| 3 | *Coccinella* and related genera | *Coccinella florissantensis* | 37.0 - 33.9 | 36.20 ± 0.1 | Cockerell, 1906 | USA (Colorado) | Chadronian |
| 4 | *Chilocorus* crown | *Chilocorus inflatus* | 33.9 - 27.3 | 30.5 ± 0.3 | Förster, 1891 | France | Early/Lower Oligocene |
| 5 | *Scymnus* crown | *Scymnus angulatus* | 33.9 - 27.3 | 30.5 ± 0.3 | Förster, 1891 | France | Early/Lower Oligocene |
| **Refrences:** | | | | | | | |
| Förster, B. Die Insekten des" Plattigen Steinmergels" von Brunstatt. *Abhandlungen zur Geologischen Specialkarte von Elsass-Lothringen* **1891**, *3*, 335-593. | | | | | | | |
| Cockerell, T.D.A. Fossil Saw-flies from Florissant Colorado. *Bulletin of the American Museum of Natural History* **1906**, *22*, 499-501. | | | | | | | |
| Kirejtshuk, A.; Nel, A. The oldest representatives of the family Coccinellidae (Coleoptera: Polyphaga) from the lowermost Eocene Oise amber (France). *Zoosystematica Rossica* **2012**, *21*, 131-144. | | | | | | | |

| **TABLE S2 \|** Nucleotide composition and skewness of mitogenomes. | | | | | | | | | | |
| --- | --- | --- | --- | --- | --- | --- | --- | --- | --- | --- |
| **Regions** | **Species** | **Size (bp)** | **A%** | **T%** | **C%** | **G%** | **AT%** | **GC%** | **AT-skew** | **GC-skew** |
| Whole genome | *Coelophora circumvelata* | 18596 | 41.5 | 37.5 | 11.8 | 9.2 | 79 | 21 | 0.05 | -0.124 |
|  | *Harmonia dimidiata* | 19945 | 38.2 | 36.7 | 14.1 | 11 | 74.9 | 25.1 | 0.019 | -0.121 |
|  | *Harmonia yedoensis* | 17356 | 40.2 | 38.4 | 12.8 | 8.5 | 78.6 | 21.3 | 0.02 | -0.2 |
|  | *Maroilleis hauseri* | 16943 | 41 | 35.6 | 13.7 | 9.7 | 76.6 | 23.4 | 0.07 | -0.173 |
|  | *Micarspic allardi* | 17173 | 41.4 | 37.6 | 12.5 | 8.5 | 79 | 21 | 0.049 | -0.188 |
|  | *Micraspis satoi* | 15868 | 41.1 | 37.3 | 12.7 | 9 | 78.4 | 21.7 | 0.048 | -0.174 |
|  | *Propylea luteopustulata* | 14776 | 40 | 37.9 | 10.7 | 11.4 | 77.9 | 22.1 | 0.027 | 0.03 |
|  | *Singhikalia duodecimguttata* | 19843 | 41.7 | 37.3 | 11.3 | 9.7 | 79 | 21 | 0.056 | -0.074 |
|  | *Synonia consanguihae* | 18792 | 41.5 | 38.6 | 11.9 | 8 | 80.1 | 19.9 | 0.036 | -0.196 |
| PCGs | *Coelophora circumvelata* | 11055 | 33.1 | 45 | 10.8 | 11.1 | 78.1 | 21.9 | -0.152 | 0.016 |
|  | *Harmonia dimidiata* | 10992 | 32.7 | 42.9 | 12.3 | 12.1 | 75.6 | 24.4 | -0.135 | -0.008 |
|  | *Harmonia yedoensis* | 10983 | 33.3 | 44.3 | 11.4 | 11 | 77.6 | 22.4 | -0.14 | -0.02 |
|  | *Maroilleis hauseri* | 11028 | 32.2 | 42.5 | 12.8 | 12.6 | 74.7 | 25.4 | -0.138 | -0.007 |
|  | *Micarspic allardi* | 10989 | 33.2 | 44 | 11.4 | 11.5 | 77.2 | 22.9 | -0.14 | 0.006 |
|  | *Micraspis satoi* | 10992 | 32.7 | 44.5 | 11.4 | 11.4 | 77.2 | 22.8 | -0.153 | 0.002 |
|  | *Propylea luteopustulata* | 11034 | 33.7 | 45.1 | 10.3 | 11 | 78.8 | 21.3 | -0.145 | 0.032 |
|  | *Singhikalia duodecimguttata* | 9414 | 33.9 | 45.7 | 9.7 | 10.6 | 79.6 | 20.3 | -0.148 | 0.046 |
|  | *Synonia consanguihae* | 9471 | 34.6 | 45.8 | 9.6 | 10 | 80.4 | 19.6 | -0.138 | 0.018 |
| tRNAs | *Coelophora circumvelata* | 1437 | 40.8 | 39.7 | 8.2 | 11.3 | 80.5 | 19.5 | 0.014 | 0.16 |
|  | *Harmonia dimidiata* | 1407 | 40.4 | 38.3 | 9.2 | 12.1 | 78.7 | 21.3 | 0.026 | 0.133 |
|  | *Harmonia yedoensis* | 1394 | 40.4 | 38.5 | 9.6 | 11.5 | 78.9 | 21.1 | 0.02 | 0.09 |
|  | *Maroilleis hauseri* | 1418 | 41.6 | 37.2 | 9 | 12.2 | 78.8 | 21.2 | 0.056 | 0.15 |
|  | *Micarspic allardi* | 1393 | 41.2 | 40 | 8 | 10.8 | 81.2 | 18.8 | 0.015 | 0.145 |
|  | *Micraspis satoi* | 1388 | 40.5 | 39.9 | 8.2 | 11.4 | 80.4 | 19.6 | 0.007 | 0.162 |
|  | *Propylea luteopustulata* | 1425 | 41.3 | 38.1 | 8.6 | 11.9 | 79.4 | 20.5 | 0.041 | 0.16 |
|  | *Singhikalia duodecimguttata* | 1403 | 41.8 | 38 | 8.8 | 11.4 | 79.8 | 20.2 | 0.048 | 0.131 |
|  | *Synonia consanguihae* | 1399 | 41.7 | 38.7 | 8.6 | 10.9 | 80.4 | 19.5 | 0.038 | 0.117 |
| rRNAs | *Coelophora circumvelata* | 2137 | 37.3 | 44.5 | 6.4 | 11.7 | 81.8 | 18.1 | -0.088 | 0.297 |
|  | *Harmonia dimidiata* | 2084 | 38.3 | 41.3 | 6.5 | 13.9 | 79.6 | 20.4 | -0.037 | 0.36 |
|  | *Harmonia yedoensis* | 2091 | 39.5 | 41.8 | 6.2 | 12.5 | 81.3 | 18.7 | -0.03 | 0.34 |
|  | *Maroilleis hauseri* | 2145 | 36.2 | 44 | 6.7 | 13.2 | 80.2 | 19.9 | -0.097 | 0.329 |
|  | *Micarspic allardi* | 1960 | 36.8 | 44.3 | 6.5 | 12.4 | 81.1 | 18.9 | -0.092 | 0.314 |
|  | *Micraspis satoi* | 2134 | 36.7 | 45 | 6 | 12.3 | 81.7 | 18.3 | -0.101 | 0.349 |
|  | *Propylea luteopustulata* | 2030 | 40 | 32.8 | 20.7 | 6.6 | 72.8 | 27.3 | 0.098 | -0.519 |
|  | *Singhikalia duodecimguttata* | 2039 | 38.9 | 43.1 | 6.2 | 11.9 | 82 | 18.1 | -0.051 | 0.315 |
|  | *Synonia consanguihae* | 2029 | 38.9 | 43 | 6.2 | 11.9 | 81.9 | 18.1 | -0.05 | 0.315 |
| 1st codon position | *Coelophora circumvelata* | 3685 | 35.8 | 37.8 | 10.1 | 16.3 | 73.6 | 26.4 | -0.027 | 0.234 |
|  | *Harmonia dimidiata* | 3664 | 35.5 | 36.2 | 10.9 | 17.4 | 71.7 | 28.3 | -0.01 | 0.23 |
|  | *Harmonia yedoensis* | 3661 | 35.2 | 37.1 | 11 | 16.7 | 72.3 | 27.7 | -0.03 | 0.21 |
|  | *Maroilleis hauseri* | 3676 | 34.8 | 36.1 | 11.6 | 17.5 | 70.9 | 29.1 | -0.019 | 0.203 |
|  | *Micarspic allardi* | 3663 | 36.3 | 36.6 | 10.1 | 17 | 72.9 | 27.1 | -0.005 | 0.253 |
|  | *Micraspis satoi* | 3664 | 36.5 | 36.7 | 10.4 | 16.3 | 73.2 | 26.7 | -0.003 | 0.221 |
|  | *Propylea luteopustulata* | 3678 | 36.7 | 37.4 | 9.5 | 16.4 | 74.1 | 25.9 | -0.01 | 0.264 |
|  | *Singhikalia duodecimguttata* | 3138 | 35.9 | 39.1 | 9.2 | 15.7 | 75 | 24.9 | -0.042 | 0.263 |
|  | *Synonia consanguihae* | 3157 | 37.3 | 38.3 | 9.2 | 15.2 | 75.6 | 24.4 | -0.014 | 0.244 |
| 2nd codon position | *Coelophora circumvelata* | 3685 | 21.2 | 49.1 | 16.9 | 12.8 | 70.3 | 29.7 | -0.396 | -0.14 |
|  | *Harmonia dimidiata* | 3664 | 21.3 | 48.2 | 17.4 | 13.1 | 69.5 | 30.5 | -0.388 | -0.142 |
|  | *Harmonia yedoensis* | 3661 | 21.4 | 48.8 | 16.9 | 12.8 | 70.2 | 29.7 | -0.39 | -0.14 |
|  | *Maroilleis hauseri* | 3676 | 21.5 | 48.2 | 17.3 | 13.1 | 69.7 | 30.4 | -0.383 | -0.137 |
|  | *Micarspic allardi* | 3663 | 21.7 | 49.1 | 17 | 12.2 | 70.8 | 29.2 | -0.387 | -0.166 |
|  | *Micraspis satoi* | 3664 | 21.6 | 49.4 | 16.6 | 12.4 | 71 | 29 | -0.392 | -0.145 |
|  | *Propylea luteopustulata* | 3678 | 21.6 | 49.3 | 16.3 | 12.8 | 70.9 | 29.1 | -0.39 | -0.121 |
|  | *Singhikalia duodecimguttata* | 3138 | 22 | 49.9 | 15.6 | 12.5 | 71.9 | 28.1 | -0.388 | -0.112 |
|  | *Synonia consanguihae* | 3157 | 22.3 | 50 | 15.5 | 12.3 | 72.3 | 27.8 | -0.383 | -0.114 |
| **TABLE S2 \|** *Cont.* | | | | | | | | | | |
| 3rd codon position | *Coelophora circumvelata* | 3685 | 42.4 | 48.1 | 5.2 | 4.2 | 90.5 | 9.4 | -0.063 | -0.106 |
|  | *Harmonia dimidiata* | 3664 | 41.3 | 44.2 | 8.6 | 5.9 | 85.5 | 14.5 | -0.034 | -0.19 |
|  | *Harmonia yedoensis* | 3661 | 43.4 | 47 | 6.3 | 3.3 | 90.4 | 9.6 | -0.04 | -0.31 |
|  | *Maroilleis hauseri* | 3676 | 40.3 | 43.2 | 9.4 | 7.1 | 83.5 | 16.5 | -0.036 | -0.137 |
|  | *Micarspic allardi* | 3663 | 41.5 | 46.2 | 6.9 | 5.4 | 87.7 | 12.3 | -0.053 | -0.126 |
|  | *Micraspis satoi* | 3664 | 40 | 47.4 | 7.1 | 5.5 | 87.4 | 12.6 | -0.084 | -0.123 |
|  | *Propylea luteopustulata* | 3678 | 42.7 | 48.5 | 5 | 3.8 | 91.2 | 8.8 | -0.064 | -0.142 |
|  | *Singhikalia duodecimguttata* | 3138 | 43.8 | 48.2 | 4.3 | 3.7 | 92 | 8 | -0.047 | -0.075 |
|  | *Synonia consanguihae* | 3157 | 44.3 | 49 | 4.2 | 2.5 | 93.3 | 6.7 | -0.05 | -0.257 |
| Control region | *Coelophora circumvelata* | 4004 | 40.9 | 38.2 | 9.9 | 11.1 | 79.1 | 20.9 | 0.034 | 0.057 |
|  | *Harmonia dimidiata* | 5442 | 34.8 | 36 | 13.6 | 15.6 | 70.8 | 29.2 | -0.017 | 0.068 |
|  | *Harmonia yedoensis* | 2862 | 40.5 | 39.4 | 11.9 | 8.2 | 79.9 | 20.1 | 0.014 | -0.184 |
|  | *Maroilleis hauseri* | 1716 | 44.2 | 38.9 | 10.5 | 6.4 | 83.1 | 16.9 | 0.064 | -0.243 |
|  | *Micarspic allardi* | 2690 | 43.6 | 39.6 | 9.5 | 7.3 | 83.2 | 16.8 | 0.048 | -0.131 |
|  | *Micraspis satoi* | 1348 | 42.1 | 37.5 | 10.7 | 9.6 | 79.6 | 20.4 | 0.058 | -0.054 |
|  | *Propylea luteopustulata* | 1676 | 25.7 | 44.3 | 0.6 | 29.3 | 70 | 30 | -0.266 | 0.957 |
|  | *Singhikalia duodecimguttata* | 5318 | 42.3 | 35.8 | 9.1 | 12.8 | 78.1 | 21.9 | 0.083 | 0.169 |
|  | *Synonia consanguihae* | 4273 | 41.7 | 39.2 | 11.9 | 7.3 | 80.9 | 19.1 | 0.031 | -0.241 |

| TABLE S3 \| Annotation and organization of *Coelophora circumvelata* mitochondrial genomes. | | | | | | | |
| --- | --- | --- | --- | --- | --- | --- | --- |
| Feature | **Strand** | **Position** | **Length (bp)** | **Initiation codon** | **Stop codon** | **Anticodon** | **Intergenic nucleotide** |
| *trnI* | J | 1-63 | 63 |  |  | GAT | 32 |
| *trnQ* | N | 96-164 | 69 |  |  | TTG | -2 |
| *trnM* | J | 163-233 | 71 |  |  | CAT | 6 |
| *nad2* | J | 240-1241 | 1002 | ATC | TAA |  | -2 |
| *trnW* | J | 1240-1306 | 67 |  |  | TCA | -8 |
| *trnC* | N | 1299-1361 | 63 |  |  | GCA | 0 |
| *trnY* | N | 1362-1426 | 65 |  |  | GTA | -2 |
| *cox1* | J | 1425-2966 | 1542 | TCG | TAA |  | -5 |
| *trnL2* | J | 2962-3025 | 64 |  |  | TAA | 0 |
| *cox2* | J | 3026-3707 | 682 | ATT | T |  | -3 |
| *trnK* | J | 3705-3773 | 69 |  |  | CTT | 5 |
| *trnD* | J | 3779-3843 | 65 |  |  | GTC | 0 |
| *atp8* | J | 3844-3996 | 153 | ATT | TAA |  | -7 |
| *atp6* | J | 3990-4645 | 656 | ATG | TA |  | 1 |
| *cox3* | J | 4647-5427 | 781 | ATG | T |  | 0 |
| *trnG* | J | 5428-5489 | 62 |  |  | TCC | -3 |
| *nad3* | J | 5487-5837 | 351 | ATA | TAG |  | -2 |
| *trnA* | J | 5836-5898 | 63 |  |  | TGC | 0 |
| *trnR* | J | 5899-5967 | 69 |  |  | TCG | 0 |
| *trnN* | J | 5968-6032 | 65 |  |  | GTT | 0 |
| *trnS1* | J | 6033-6090 | 58 |  |  | TCT | 2 |
| *trnE* | J | 6093-6155 | 63 |  |  | TTC | -2 |
| *trnF* | N | 6154-6217 | 64 |  |  | GAA | -1 |
| *nad5* | N | 6217-7934 | 1718 | ATA | TA |  | -3 |
| *trnH* | N | 7932-7994 | 63 |  |  | GTG | -1 |
| *nad4* | N | 7994-9315 | 1322 | ATG | TA |  | -7 |
| *nad4l* | N | 9309-9587 | 279 | ATG | TAA |  | 0 |
| *trnT* | J | 9588-9654 | 67 |  |  | TGT | 0 |
| *trnP* | N | 9655-9715 | 61 |  |  | TGG | 2 |
| *nad6* | J | 9718-10211 | 494 | ATA | TA |  | -1 |
| *cytb* | J | 10211-11350 | 1140 | ATG | TAA |  | -2 |
| *trnS2* | J | 11349-11413 | 65 |  |  | TGA | 17 |
| *nad1* | N | 11431-12375 | 945 | ATA | TAG |  | -3 |
| *trnL1* | N | 12373-12445 | 73 |  |  | TAG | -49 |
| *rrnL* | N | 12397-13735 | 1339 |  |  |  | -7 |
| *trnV* | N | 13729-13796 | 68 |  |  | TAC | -2 |
| *rrnS* | N | 13795-14592 | 798 |  |  |  | 0 |
| CR | J | 14593-18596 | 4004 |  |  |  | 0 |

| TABLE S4 \| Annotation and organization of *Harmonia dimidiate* mitochondrial genomes. | | | | | | | |
| --- | --- | --- | --- | --- | --- | --- | --- |
| Feature | **Strand** | **Position** | **Length (bp)** | **Initiation codon** | **Stop codon** | **Anticodon** | **Intergenic nucleotide** |
| *trnI* | J | 1-64 | 64 |  |  | GAT | 30 |
| *trnQ* | N | 95-162 | 68 |  |  | TTG | -1 |
| *trnM* | J | 162-228 | 67 |  |  | CAT | 6 |
| *nad2* | J | 235-1233 | 999 | ATT | TAA |  | -2 |
| *trnW* | J | 1232-1295 | 64 |  |  | TCA | -8 |
| *trnC* | N | 1288-1349 | 62 |  |  | GCA | 0 |
| *trnY* | N | 1350-1413 | 64 |  |  | GTA | 34 |
| *cox1* | J | 1448-2953 | 1542 | ATT | TAA |  | -5 |
| *trnL2* | J | 2949-3013 | 65 |  |  | TAA | 0 |
| *cox2* | J | 3014-3689 | 676 | ATC | T |  | -3 |
| *trnK* | J | 3687-3756 | 70 |  |  | CTT | 0 |
| *trnD* | J | 3757-3820 | 64 |  |  | GTC | 0 |
| *atp8* | J | 3821-3973 | 153 | ATC | TAA |  | -7 |
| *atp6* | J | 3967-4624 | 658 | ATG | T |  | 0 |
| *cox3* | J | 4625-5405 | 781 | ATG | T |  | 0 |
| *trnG* | J | 5406-5469 | 64 |  |  | TCC | -3 |
| *nad3* | J | 5467-5823 | 357 | ATA | TAG |  | -2 |
| *trnA* | J | 5822-5883 | 62 |  |  | TGC | 0 |
| *trnR* | J | 5884-5945 | 62 |  |  | TCG | 0 |
| *trnN* | J | 5946-6009 | 64 |  |  | GTT | 0 |
| *trnS1* | J | 6010-6064 | 55 |  |  | TCT | 1 |
| *trnE* | J | 6066-6128 | 63 |  |  | TTC | -2 |
| *trnF* | N | 6127-6192 | 66 |  |  | GAA | -1 |
| *nad5* | N | 6192-7906 | 1718 | ATT | TA |  | 0 |
| *trnH* | N | 7907-7968 | 62 |  |  | GTG | -1 |
| *nad4* | N | 7968-9292 | 1325 | ATG | TA |  | -7 |
| *nad4l* | N | 9286-9543 | 267 | ATA | TAA |  | 21 |
| *trnT* | J | 9565-9629 | 65 |  |  | TGT | 0 |
| *trnP* | N | 9630-9690 | 61 |  |  | TGG | 2 |
| *nad6* | J | 9693-10177 | 485 | ATA | TA |  | -1 |
| *cytb* | J | 10177-11316 | 1140 | ATG | TAA |  | -2 |
| *trnS2* | J | 11315-11379 | 65 |  |  | TGA | 17 |
| *nad1* | N | 11397-12344 | 948 | ATA | TAG |  | -3 |
| *trnL1* | N | 12342-12408 | 64 |  |  | TAG | -43 |
| *rrnL* | N | 12366-13701 | 1336 |  |  |  | -7 |
| *trnV* | N | 13695-13757 | 63 |  |  | TAC | -2 |
| *rrnS* | N | 13756-14503 | 748 |  |  |  | 0 |
| CR | J | 14504-19945 | 5442 |  |  |  | 0 |

| TABLE S5 \| Annotation and organization of *Harmonia* *yedoensis* mitochondrial genomes. | | | | | | | |
| --- | --- | --- | --- | --- | --- | --- | --- |
| Feature | **Strand** | **Position** | **Length (bp)** | **Initiation codon** | **Stop codon** | **Anticodon** | **Intergenic nucleotide** |
| *trnI* | J | 1-57 | 57 |  |  | GAT | 26 |
| *trnQ* | N | 84-151 | 68 |  |  | TTG | 0 |
| *trnM* | J | 152-218 | 67 |  |  | CAT | 9 |
| *nad2* | J | 228-1223 | 996 | ATA | TAA |  | -2 |
| *trnW* | J | 1222-1284 | 63 |  |  | TCA | -8 |
| *trnC* | N | 1277-1338 | 62 |  |  | GCA | 0 |
| *trnY* | N | 1339-1400 | 62 |  |  | GTA | 34 |
| *cox1* | J | 1435-2940 | 1506 | ATC | TAA |  | -5 |
| *trnL2* | J | 2936-3000 | 65 |  |  | TAA | 0 |
| *cox2* | J | 3001-3676 | 676 | ATA | T |  | -3 |
| *trnK* | J | 3674-3743 | 70 |  |  | CTT | 0 |
| *trnD* | J | 3744-3806 | 63 |  |  | GTC | 0 |
| *atp8* | J | 3807-3959 | 153 | ATT | TAA |  | -7 |
| *atp6* | J | 3953-4610 | 658 | ATG | T |  | 0 |
| *cox3* | J | 4611-5391 | 781 | ATG | T |  | 0 |
| *trnG* | J | 5392-5453 | 62 |  |  | TCC | -3 |
| *nad3* | J | 5451-5807 | 357 | ATA | TAG |  | -2 |
| *trnA* | J | 5806-5867 | 62 |  |  | TGC | 0 |
| *trnR* | J | 5868-5928 | 61 |  |  | TCG | 0 |
| *trnN* | J | 5929-5993 | 65 |  |  | GTT | 0 |
| *trnS1* | J | 5994-6047 | 54 |  |  | TCT | 1 |
| *trnE* | J | 6049-6111 | 63 |  |  | TTC | -2 |
| *trnF* | N | 6110-6174 | 65 |  |  | GAA | -1 |
| *nad5* | N | 6174-7888 | 1715 | ATA | TA |  | 0 |
| *trnH* | N | 7889-7951 | 63 |  |  | GTG | -1 |
| *nad4* | N | 7951-9272 | 1322 | ATG | TA |  | -7 |
| *nad4l* | N | 9266-9517 | 252 | ATT | TAA |  | 27 |
| *trnT* | J | 9545-9609 | 65 |  |  | TGT | 0 |
| *trnP* | N | 9610-9672 | 63 |  |  | TGG | 2 |
| *nad6* | J | 9675-10165 | 491 | ATC | TA |  | -1 |
| *cytb* | J | 10165-11304 | 1140 | ATG | TAA |  | -2 |
| *trnS2* | J | 11303-11367 | 65 |  |  | TGA | 17 |
| *nad1* | N | 11385-12329 | 945 | ATA | TAG |  | -3 |
| *trnL1* | N | 12327-12393 | 67 |  |  | TAG | -43 |
| *rrnL* | N | 12351-13694 | 1344 |  |  |  | -7 |
| *trnV* | N | 13688-13749 | 62 |  |  | TAC | -2 |
| *rrnS* | N | 13748-14494 | 747 |  |  |  | 0 |
| CR | J | 14495-17356 | 2862 |  |  |  | 0 |

| TABLE S6 \| Annotation and organization of *Maroilleis hauseri* mitochondrial genomes. | | | | | | | |
| --- | --- | --- | --- | --- | --- | --- | --- |
| Feature | **Strand** | **Position** | **Length (bp)** | **Initiation codon** | **Stop codon** | **Anticodon** | **Intergenic nucleotide** |
| *trnI* | J | 1-68 | 68 |  |  | GAT | 640 |
| *trnQ* | N | 709-777 | 69 |  |  | TTG | -3 |
| *trnM* | J | 775-842 | 68 |  |  | CAT | -3 |
| *nad2* | J | 840-1850 | 1011 | ATA | TAA |  | -2 |
| *trnW* | J | 1849-1913 | 65 |  |  | TCA | -8 |
| *trnC* | N | 1906-1968 | 63 |  |  | GCA | 0 |
| *trnY* | N | 1969-2033 | 65 |  |  | GTA | 34 |
| *cox1* | J | 2068-3573 | 1506 | ATT | TAA |  | -5 |
| *trnL2* | J | 3569-3632 | 64 |  |  | TAA | 0 |
| *cox2* | J | 3633-4311 | 679 | ATT | T |  | 0 |
| *trnK* | J | 4312-4381 | 70 |  |  | CTT | 0 |
| *trnD* | J | 4382-4446 | 65 |  |  | GTC | 0 |
| *atp8* | J | 4447-4599 | 153 | ATC | TAA |  | -7 |
| *atp6* | J | 4593-5251 | 659 | ATG | TA |  | -1 |
| *cox3* | J | 5251-6032 | 782 | ATG | TA |  | -1 |
| *trnG* | J | 6032-6094 | 63 |  |  | TCC | -3 |
| *nad3* | J | 6092-6448 | 357 | ATA | TAG |  | -2 |
| *trnA* | J | 6447-6509 | 63 |  |  | TGC | 2 |
| *trnR* | J | 6512-6574 | 63 |  |  | TCG | 3 |
| *trnN* | J | 6578-6641 | 64 |  |  | GTT | 0 |
| *trnS1* | J | 6642-6697 | 56 |  |  | TCT | 4 |
| *trnE* | J | 6702-6766 | 65 |  |  | TTC | 6 |
| *trnF* | N | 6773-6836 | 64 |  |  | GAA | -1 |
| *nad5* | N | 6836-8553 | 1718 | ATA | TA |  | -3 |
| *trnH* | N | 8551-8614 | 64 |  |  | GTG | -1 |
| *nad4* | N | 8614-9935 | 1322 | ATG | TA |  | -7 |
| *nad4l* | N | 9929-10201 | 273 | ATT | TAA |  | 6 |
| *trnT* | J | 10208-10272 | 65 |  |  | TGT | 0 |
| *trnP* | N | 10273-10335 | 63 |  |  | TGG | 2 |
| *nad6* | J | 10338-10831 | 494 | ATA | TA |  | -1 |
| *cytb* | J | 10831-11970 | 1140 | ATG | TAA |  | -2 |
| *trnS2* | J | 11969-12031 | 63 |  |  | TGA | 16 |
| *nad1* | N | 12048-12992 | 945 | ATA | TAG |  | -3 |
| *trnL1* | N | 12990-13051 | 62 |  |  | TAG | -38 |
| *rrnL* | N | 13014-14342 | 1329 |  |  |  | 3 |
| *trnV* | N | 14346-14411 | 66 |  |  | TAC | 0 |
| *rrnS* | N | 14412-15227 | 816 |  |  |  | 0 |
| CR | J | 15228-16943 | 1716 |  |  |  | 0 |

| TABLE S7 \| Annotation and organization of *Micarspic allardi* mitochondrial genomes. | | | | | | | |
| --- | --- | --- | --- | --- | --- | --- | --- |
| Feature | **Strand** | **Position** | **Length (bp)** | **Initiation codon** | **Stop codon** | **Anticodon** | **Intergenic nucleotide** |
| *trnI* | J | 1-67 | 67 |  |  | GAT | 63 |
| *trnQ* | N | 131-199 | 69 |  |  | TTG | -2 |
| *trnM* | J | 198-264 | 67 |  |  | CAT | 15 |
| *nad2* | J | 280-1268 | 989 | ATT | TA |  | 2 |
| *trnW* | J | 1271-1333 | 63 |  |  | TCA | -8 |
| *trnC* | N | 1326-1385 | 60 |  |  | GCA | 3 |
| *trnY* | N | 1389-1447 | 59 |  |  | GTA | 34 |
| *cox1* | J | 1482-2970 | 1489 | ATC | TA |  | 16 |
| *trnL2* | J | 2987-3045 | 59 |  |  | TAA | 0 |
| *cox2* | J | 3046-3724 | 679 | ATT | T |  | 0 |
| *trnK* | J | 3725-3795 | 71 |  |  | CTT | -1 |
| *trnD* | J | 3795-3858 | 64 |  |  | GTC | 0 |
| *atp8* | J | 3859-4011 | 153 | ATA | TAA |  | -7 |
| *atp6* | J | 4005-4661 | 657 | ATG | TAA |  | 0 |
| *cox3* | J | 4662-5442 | 781 | ATG | T |  | 0 |
| *trnG* | J | 5443-5504 | 62 |  |  | TCC | 0 |
| *nad3* | J | 5505-5858 | 354 | ATA | TAG |  | -2 |
| *trnA* | J | 5857-5918 | 62 |  |  | TGC | -1 |
| *trnR* | J | 5918-5978 | 61 |  |  | TCG | 0 |
| *trnN* | J | 5979-6038 | 60 |  |  | GTT | 0 |
| *trnS1* | J | 6039-6092 | 54 |  |  | TCT | 0 |
| *trnE* | J | 6093-6190 | 98 |  |  | TTC | -1 |
| *trnF* | N | 6190-6252 | 63 |  |  | GAA | 0 |
| *nad5* | N | 6253-7960 | 1708 | ATT | T |  | 0 |
| *trnH* | N | 7961-8022 | 62 |  |  | GTG | 0 |
| *nad4* | N | 8023-9343 | 1321 | ATG | T |  | -7 |
| *nad4l* | N | 9337-9615 | 279 | ATG | TAA |  | 1 |
| *trnT* | J | 9617-9678 | 62 |  |  | TGT | 0 |
| *trnP* | N | 9679-9739 | 61 |  |  | TGG | 2 |
| *nad6* | J | 9742-10242 | 501 | ATC | TAA |  | -26 |
| *cytb* | J | 10217-11356 | 1140 | ATG | TAA |  | -2 |
| *trnS2* | J | 11355-11401 | 47 |  |  | TGA | 32 |
| *nad1* | N | 11434-12378 | 945 | ATA | TAG |  | 0 |
| *trnL1* | N | 12379-12439 | 61 |  |  | TAG | 0 |
| *rrnL* | N | 12440-13702 | 1263 |  |  |  | 24 |
| *trnV* | N | 13727-13787 | 61 |  |  | TAC | -1 |
| *rrnS* | N | 13787-14483 | 697 |  |  |  | 0 |
| CR | J | 14484-17173 | 2690 |  |  |  | 0 |

| TABLE S8 \| Annotation and organization of *Micraspis satoi* mitochondrial genomes. | | | | | | | |
| --- | --- | --- | --- | --- | --- | --- | --- |
| Feature | **Strand** | **Position** | **Length (bp)** | **Initiation codon** | **Stop codon** | **Anticodon** | **Intergenic nucleotide** |
| *trnI* | J | 1-63 | 63 |  |  | GAT | 32 |
| *trnQ* | N | 96-164 | 69 |  |  | TTG | -2 |
| *trnM* | J | 163-229 | 67 |  |  | CAT | 9 |
| *nad2* | J | 239-1237 | 999 | ATA | TAA |  | -2 |
| *trnW* | J | 1236-1298 | 63 |  |  | TCA | -8 |
| *trnC* | N | 1291-1350 | 60 |  |  | GCA | 0 |
| *trnY* | N | 1351-1411 | 61 |  |  | GTA | 34 |
| *cox1* | J | 1446-2951 | 1506 | ATT | TAA |  | -5 |
| *trnL2* | J | 2947-3009 | 63 |  |  | TAA | 0 |
| *cox2* | J | 3010-3691 | 682 | ATT | T |  | -3 |
| *trnK* | J | 3689-3758 | 70 |  |  | CTT | 0 |
| *trnD* | J | 3759-3821 | 63 |  |  | GTC | 0 |
| *atp8* | J | 3822-3974 | 153 | ATT | TAA |  | -7 |
| *atp6* | J | 3968-4623 | 656 | ATG | TA |  | 1 |
| *cox3* | J | 4625-5405 | 781 | ATG | T |  | 0 |
| *trnG* | J | 5406-5467 | 62 |  |  | TCC | -3 |
| *nad3* | J | 5465-5821 | 357 | ATA | TAG |  | -2 |
| *trnA* | J | 5820-5880 | 61 |  |  | TGC | 0 |
| *trnR* | J | 5881-5939 | 59 |  |  | TCG | 0 |
| *trnN* | J | 5940-6002 | 63 |  |  | GTT | 0 |
| *trnS1* | J | 6003-6055 | 53 |  |  | TCT | 1 |
| *trnE* | J | 6057-6123 | 67 |  |  | TTC | -2 |
| *trnF* | N | 6122-6182 | 61 |  |  | GAA | -1 |
| *nad5* | N | 6182-7893 | 1712 | ATT | TA |  | -3 |
| *trnH* | N | 7891-7952 | 62 |  |  | GTG | -1 |
| *nad4* | N | 7952-9273 | 1322 | ATG | TA |  | -7 |
| *nad4l* | N | 9267-9542 | 276 | ATA | TAA |  | 4 |
| *trnT* | J | 9547-9608 | 62 |  |  | TGT | 0 |
| *trnP* | N | 9609-9669 | 61 |  |  | TGG | 2 |
| *nad6* | J | 9672-10147 | 476 | ATT | TA |  | -1 |
| *cytb* | J | 10147-11286 | 1140 | ATG | TAA |  | -2 |
| *trnS2* | J | 11285-11346 | 62 |  |  | TGA | 17 |
| *nad1* | N | 11364-12305 | 942 | ATA | TAG |  | -3 |
| *trnL1* | N | 12303-12368 | 66 |  |  | TAG | -43 |
| *rrnL* | N | 12326-13654 | 1329 |  |  |  | -7 |
| *trnV* | N | 13648-13717 | 70 |  |  | TAC | -2 |
| *rrnS* | N | 13716-14520 | 805 |  |  |  | 0 |
| CR | J | 14521-15868 | 1348 |  |  |  | 0 |

| TABLE S9 \| Annotation and organization of *Propylea luteopustulata* mitochondrial genomes. | | | | | | | |
| --- | --- | --- | --- | --- | --- | --- | --- |
| Feature | **Strand** | **Position** | **Length (bp)** | **Initiation codon** | **Stop codon** | **Anticodon** | **Intergenic nucleotide** |
| *trnI* | J | 1-66 | 66 |  |  | GAT | 65 |
| *trnQ* | N | 132-199 | 68 |  |  | TTG | -2 |
| *trnM* | J | 198-268 | 71 |  |  | CAT | 15 |
| *nad2* | J | 284-1268 | 985 | ATA | T |  | 3 |
| *trnW* | J | 1272-1334 | 63 |  |  | TCA | -8 |
| *trnC* | N | 1327-1389 | 63 |  |  | GCA | 6 |
| *trnY* | N | 1396-1459 | 64 |  |  | GTA | 34 |
| *cox1* | J | 1494-2999 | 1506 | ATT | TAA |  | -1 |
| *trnL2* | J | 2999-3058 | 60 |  |  | TAA | 0 |
| *cox2* | J | 3059-3737 | 679 | ATT | T |  | 0 |
| *trnK* | J | 3738-3807 | 70 |  |  | CTT | -1 |
| *trnD* | J | 3807-3872 | 66 |  |  | GTC | 0 |
| *atp8* | J | 3873-4031 | 159 | ATT | TAA |  | -7 |
| *atp6* | J | 4025-4681 | 657 | ATG | TAA |  | 1 |
| *cox3* | J | 4683-5463 | 781 | ATG | T |  | 0 |
| *trnG* | J | 5464-5528 | 65 |  |  | TCC | 0 |
| *nad3* | J | 5529-5882 | 354 | ATT | TAA |  | -2 |
| *trnA* | J | 5881-5944 | 64 |  |  | TGC | -1 |
| *trnR* | J | 5944-6007 | 64 |  |  | TCG | 0 |
| *trnN* | J | 6008-6071 | 64 |  |  | GTT | 0 |
| *trnS1* | J | 6072-6129 | 58 |  |  | TCT | 0 |
| *trnE* | J | 6130-6192 | 63 |  |  | TTC | -1 |
| *trnF* | N | 6192-6259 | 68 |  |  | GAA | -1 |
| *nad5* | N | 6259-7974 | 1716 | ATT | TAA |  | 0 |
| *trnH* | N | 7975-8041 | 67 |  |  | GTG | 0 |
| *nad4* | N | 8042-9362 | 1321 | ATG | T |  | -7 |
| *nad4l* | N | 9356-9634 | 279 | ATG | TAG |  | 1 |
| *trnT* | J | 9636-9703 | 68 |  |  | TGT | 0 |
| *trnP* | N | 9704-9764 | 61 |  |  | TGG | 2 |
| *nad6* | J | 9767-10285 | 519 | ATA | TAA |  | -20 |
| *cytb* | J | 10266-11405 | 1140 | ATG | TAA |  | 2 |
| *trnS2* | J | 11408-11472 | 65 |  |  | TGA | 18 |
| *nad1* | N | 11491-12432 | 942 | ATT | TAG |  | 0 |
| *trnL1* | N | 12433-12494 | 62 |  |  | TAG | 0 |
| *rrnL* | N | 12495-13777 | 1283 |  |  |  | 21 |
| *trnV* | N | 13799-13863 | 65 |  |  | TAC | -1 |
| *rrnS* | N | 13863-14609 | 747 |  |  |  | 0 |
| CR | J | 14610-14776 | 1676 |  |  |  | 0 |

| TABLE S10 \| Annotation and organization of *Singhikalia duodecimguttata* mitochondrial genomes. | | | | | | | |
| --- | --- | --- | --- | --- | --- | --- | --- |
| Feature | **Strand** | **Position** | **Length (bp)** | **Initiation codon** | **Stop codon** | **Anticodon** | **Intergenic nucleotide** |
| *trnI* | J | 1-64 | 64 |  |  | GAT | 39 |
| *trnQ* | N | 104-171 | 68 |  |  | TTG | -1 |
| *trnM* | J | 171-237 | 67 |  |  | CAT | 0 |
| *nad2* | J | 238-1231 | 994 | ATC | T |  | 12 |
| *trnW* | J | 1244-1307 | 64 |  |  | TCA | -8 |
| *trnC* | N | 1300-1364 | 65 |  |  | GCA | 0 |
| *trnY* | N | 1365-1428 | 64 |  |  | GTA | 34 |
| *cox1* | J | 1463-2968 | 1506 | ATT | TAA |  | -5 |
| *trnL2* | J | 2964-3026 | 63 |  |  | TAA | 0 |
| *cox2* | J | 3027-3705 | 679 | ATT | T |  | 0 |
| *trnK* | J | 3706-3775 | 70 |  |  | CTT | -1 |
| *trnD* | J | 3775-3839 | 65 |  |  | GTC | 0 |
| *atp8* | J | 3840-3992 | 153 | ATC | TAG |  | -7 |
| *atp6* | J | 3986-4642 | 657 | ATG | TAA |  | 1 |
| *cox3* | J | 4644-5424 | 781 | ATG | T |  | 0 |
| *trnG* | J | 5425-5488 | 64 |  |  | TCC | 0 |
| *nad3* | J | 5489-5842 | 354 | ATT | TAA |  | 4 |
| *trnA* | J | 5847-5908 | 62 |  |  | TGC | -1 |
| *trnR* | J | 5908-5974 | 67 |  |  | TCG | 0 |
| *trnN* | J | 5975-6036 | 62 |  |  | GTT | 0 |
| *trnS1* | J | 6037-6091 | 55 |  |  | TCT | 0 |
| *trnE* | J | 6092-6153 | 62 |  |  | TTC | -2 |
| *trnF* | N | 6152-6223 | 72 |  |  | GAA | -1 |
| *nad5* | N | 6225-7923 | 1699 | ATA | T |  | 0 |
| *trnH* | N | 7924-7985 | 62 |  |  | GTG | 1 |
| *nad4* | N | 7987-9277 | 1291 | ATT | T |  | 23 |
| *nad4l* | N | 9301-9579 | 279 | ATG | TAA |  | 1 |
| *trnT* | J | 9581-9644 | 64 |  |  | TGT | 0 |
| *trnP* | N | 9645-9705 | 61 |  |  | TGG | 2 |
| *nad6* | J | 9708-10201 | 494 | ATT | TA |  | 38 |
| *cytb* | J | 10240-11340 | 1101 | ATT | TAA |  | 2 |
| *trnS2* | J | 11343-11397 | 55 |  |  | TGA | 23 |
| *nad1* | N | 11421-12361 | 941 | ATT | TA |  | 0 |
| *trnL1* | N | 12362-12425 | 64 |  |  | TAG | 0 |
| *rrnL* | N | 12426-13717 | 1292 |  |  |  | 0 |
| *trnV* | N | 13718-13780 | 63 |  |  | TAC | 0 |
| *rrnS* | N | 13780-14526 | 747 |  |  |  | -1 |
| CR | J | 14526-19843 | 5318 |  |  |  | 0 |

| TABLE S11 \| Annotation and organization of *Synonia consanguihae* mitochondrial genomes. | | | | | | | |
| --- | --- | --- | --- | --- | --- | --- | --- |
| Feature | **Strand** | **Position** | **Length (bp)** | **Initiation codon** | **Stop codon** | **Anticodon** | **Intergenic nucleotide** |
| *trnI* | J | 1-66 | 66 |  |  | GAT | 39 |
| *trnQ* | N | 106-173 | 68 |  |  | TTG | -1 |
| *trnM* | J | 173-239 | 67 |  |  | CAT | 0 |
| *nad2* | J | 240-1243 | 1004 | ATA | TA |  | 2 |
| *trnW* | J | 1246-1308 | 63 |  |  | TCA | -8 |
| *trnC* | N | 1301-1361 | 61 |  |  | GCA | 0 |
| *trnY* | N | 1362-1425 | 64 |  |  | GTA | 4 |
| *cox1* | J | 1460-2965 | 1506 | ATT | TAA |  | -5 |
| *trnL2* | J | 2961-3023 | 63 |  |  | TAA | 0 |
| *cox2* | J | 3024-3702 | 679 | ATA | T |  | 0 |
| *trnK* | J | 3703-3772 | 70 |  |  | CTT | -1 |
| *trnD* | J | 3772-3836 | 65 |  |  | GTC | 0 |
| *atp8* | J | 3837-3989 | 153 | ATT | TAA |  | -7 |
| *atp6* | J | 3983-4639 | 657 | ATG | TAA |  | 1 |
| *cox3* | J | 4641-5421 | 781 | ATG | T |  | 0 |
| *trnG* | J | 5422-5484 | 63 |  |  | TCC | 0 |
| *nad3* | J | 5485-5838 | 354 | ATA | TAG |  | -2 |
| *trnA* | J | 5837-5899 | 63 |  |  | TGC | -1 |
| *trnR* | J | 5899-5960 | 62 |  |  | TCG | 0 |
| *trnN* | J | 5961-6023 | 63 |  |  | GTT | 0 |
| *trnS1* | J | 6024-6081 | 58 |  |  | TCT | 0 |
| *trnE* | J | 6082-6145 | 64 |  |  | TTC | -3 |
| *trnF* | N | 6143-6216 | 74 |  |  | GAA | 1 |
| *nad5* | N | 6218-7922 | 1705 | ATT | T |  | 0 |
| *trnH* | N | 7923-7984 | 62 |  |  | GTG | 1 |
| *nad4* | N | 7986-9276 | 1291 | ATT | T |  | 23 |
| *nad4l* | N | 9300-9578 | 279 | ATG | TAG |  | 1 |
| *trnT* | J | 9580-9641 | 62 |  |  | TGT | 0 |
| *trnP* | N | 9642-9701 | 60 |  |  | TGG | 2 |
| *nad6* | J | 9704-10185 | 482 | ATT | TA |  | 5 |
| *cytb* | J | 10191-11324 | 1134 | ATA | TAA |  | 2 |
| *trnS2* | J | 11327-11381 | 55 |  |  | TGA | 22 |
| *nad1* | N | 11404-12365 | 962 | ATA | TA |  | 0 |
| *trnL1* | N | 12366-12428 | 63 |  |  | TAG | 0 |
| *rrnL* | N | 12429-13712 | 1284 |  |  |  | 0 |
| *trnV* | N | 13713-13775 | 63 |  |  | TAC | -1 |
| *rrnS* | N | 13775-14519 | 745 |  |  |  | 0 |
| CR | J | 14520-18792 | 4273 |  |  |  | 0 |


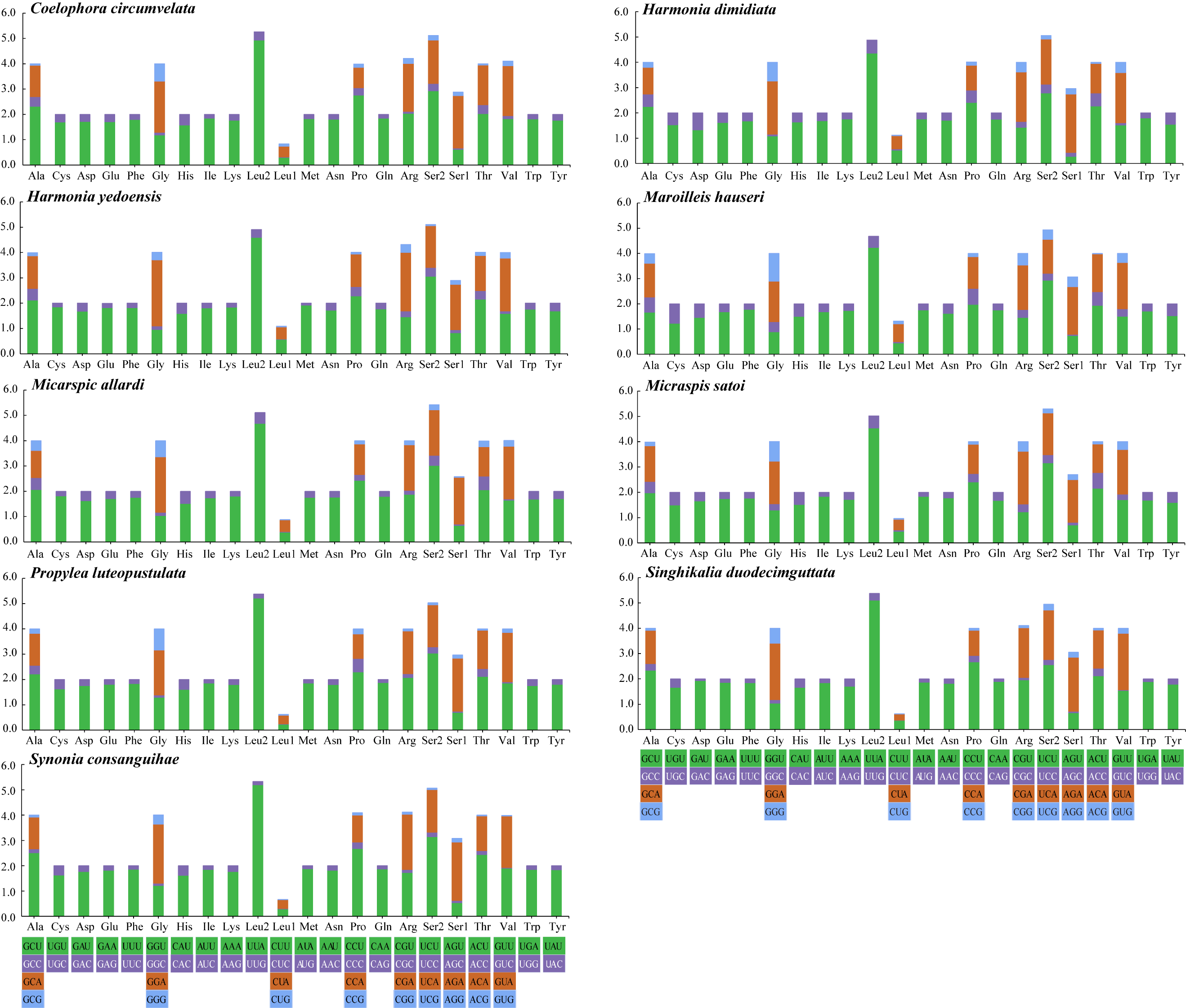


**FIGURE S1 |** Relative synonymous codon usage (RSCU) in nine newly sequenced mitogenomes.

**FIGURE S2 |** Predicted secondary cloverleaf structures for the tRNAs of newly sequence mitogenome of *Harmonia dimidiate* and *Harmonia yedoensis*.

**FIGURE S3 |** Predicted secondary cloverleaf structures for the tRNAs of newly sequence mitogenome of *Micarspic allardi, Micraspis satoi* and *Synonia consanguihae*.

**FIGURE S4 |** Predicted secondary cloverleaf structures for the tRNAs of newly sequence mitogenome of *Coelophora circumvelata*, *Maroilleis hauseri*, *Propylea luteopustulata* and *Singhikalia duodecimguttata*.


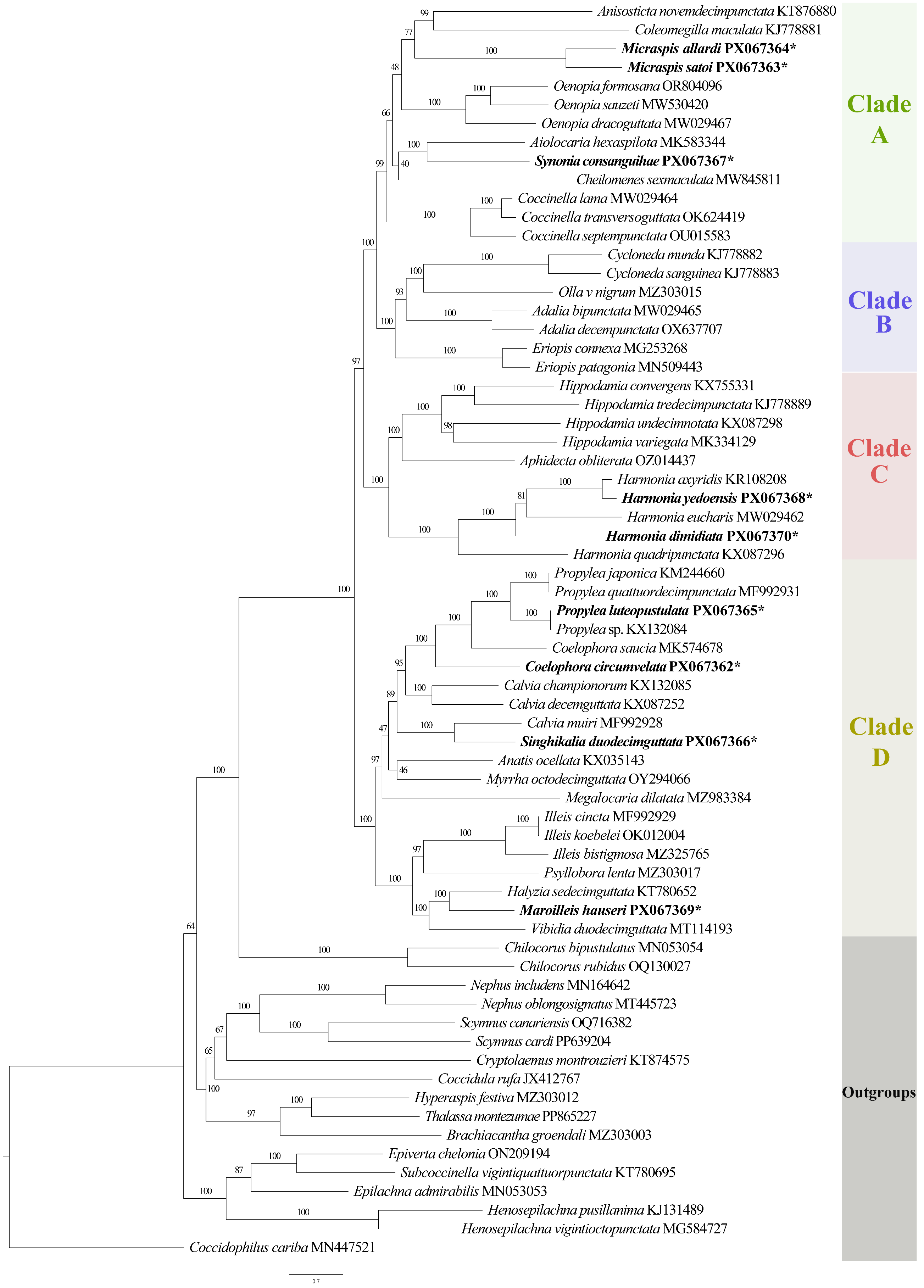


**FIGURE S5** **|** Phylogenetic tree reconstructed from PCGs_NT using Maximum likelihood method. Nodes numbers represent bootstrap values (BS). Newly sequenced species are marked with asterisks (*) and bold font.


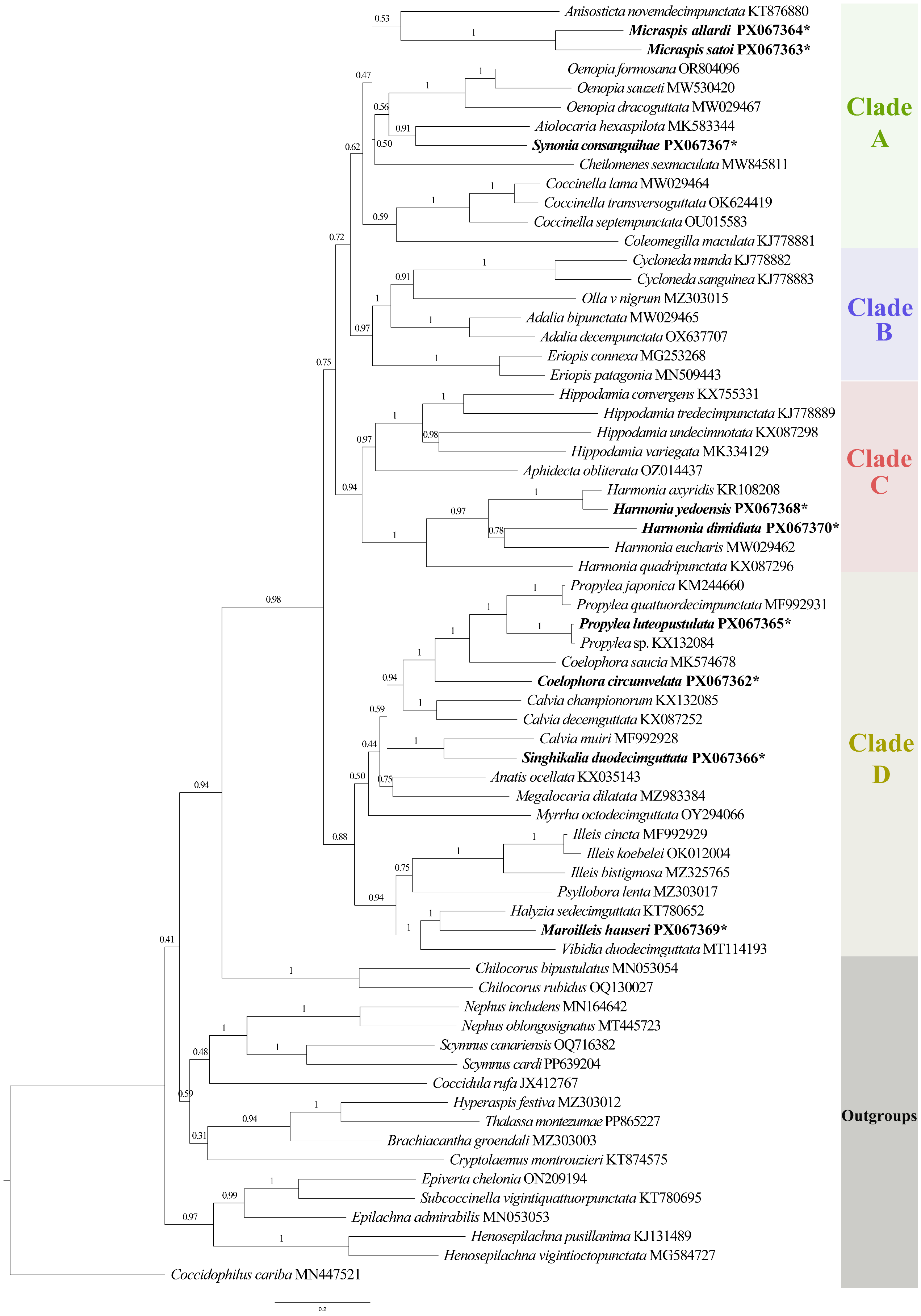


**FIGURE S6** **|** Phylogenetic tree reconstructed from PCGs_NT using Bayesian inference method. Nodes numbers represent posterior probabilities (PP). Newly sequenced species are marked with asterisks (*) and bold font.


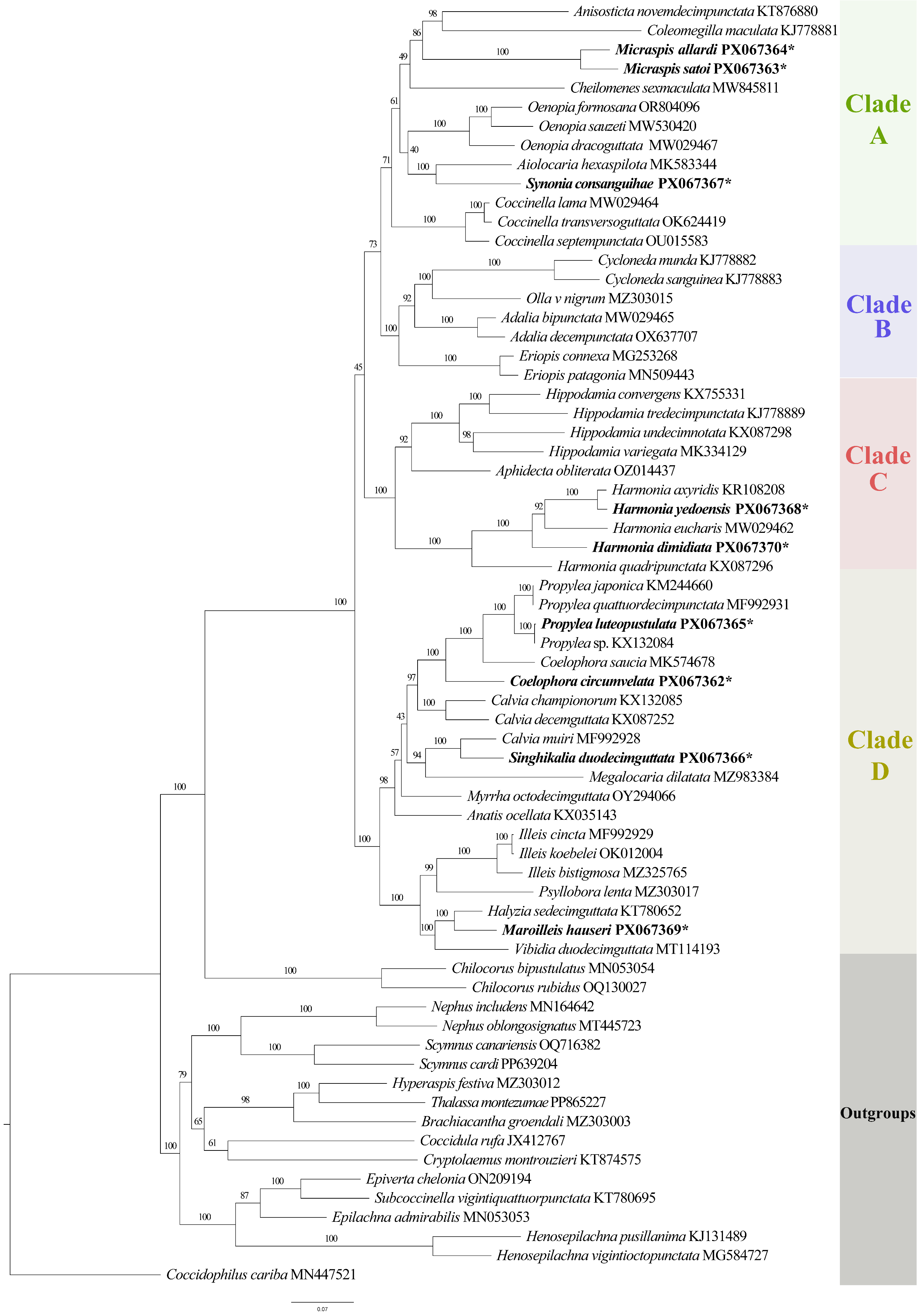


**FIGURE S7 |** Phylogenetic tree reconstructed from PCGs_AA using Maximum likelihood method. Nodes numbers represent bootstrap values (BS). Newly sequenced species are marked with asterisks (*) and bold font.


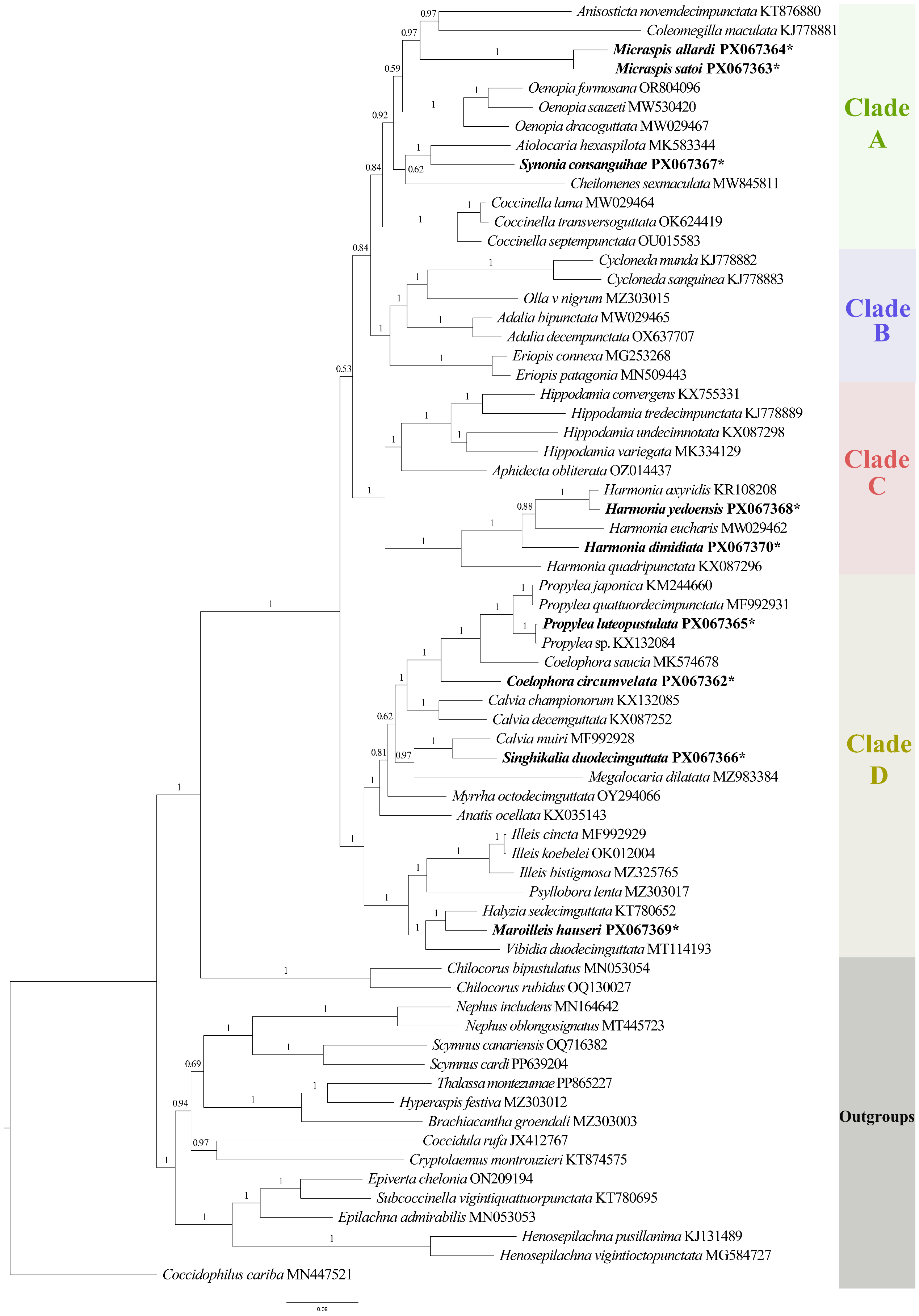


**FIGURE S8 |** Phylogenetic tree reconstructed from PCGs_AA using Bayesian inference method. Nodes numbers represent posterior probabilities (PP). Newly sequenced species are marked with asterisks (*) and bold font.
